# Limit of Blank Variability as a Determinant of Analytical Sensitivity in a DNA-Binding Dye-Based Digital Polymerase Chain Reaction for the Detection of *Chlamydia pneumoniae*

**DOI:** 10.1021/acs.analchem.6c04206

**Published:** 2026-09-04

**Authors:** Theresa Kaudela, Eveliina Taavitsainen-Wahlroos, Leena Hanski

**Affiliations:** Division of Pharmaceutical Biosciences, Faculty of Pharmacy, University of Helsinki, 00014 Helsinki, Finland

## Abstract

Digital PCR is considered a highly sensitive technique for detecting low-abundance DNA in samples containing excess background DNA. This is particularly relevant for *Chlamydia pneumoniae* infections, as this obligatory intracellular bacterium can be disseminated by phagocytes from the respiratory tract throughout the whole body. Although persistent bacteria are associated with chronic inflammatory diseases, currently, no diagnostic tools for persistent *C. pneumoniae* infections exist. In this study, an EvaGreen-based digital PCR assay targeting the chlamydial 16S rRNA gene was initially developed for the QX200 Droplet Digital PCR system; however, poor target recovery, insufficient restriction enzyme digest, and incompatibility with the DNA isolation workflow limited assay performance. Consequently, the assay was redesigned to target the chlamydial *groL* gene. Analytical performance was determined according to the Clinical and Laboratory Standards Institute (CLSI) EP17-A guideline and the ISO 20395:2019 standard. Notably, the limit of blank varied up to 4-fold between different PCR reagent lots, substantially affecting limit of detection estimates and assay interpretation at low-level. These findings demonstrate the critical importance of assay specification and reagent validation for diagnostics with DNA-binding dye-based assays. Nested PCR exhibited superior analytical sensitivity over the digital PCR assays, yet the limited throughput and increased handling complexity of nested PCR restrict its suitability for routine diagnostic applications. Overall, this study highlights important practical limitations and validation requirements for highly sensitive molecular diagnostic assays.

## Graphical abstract

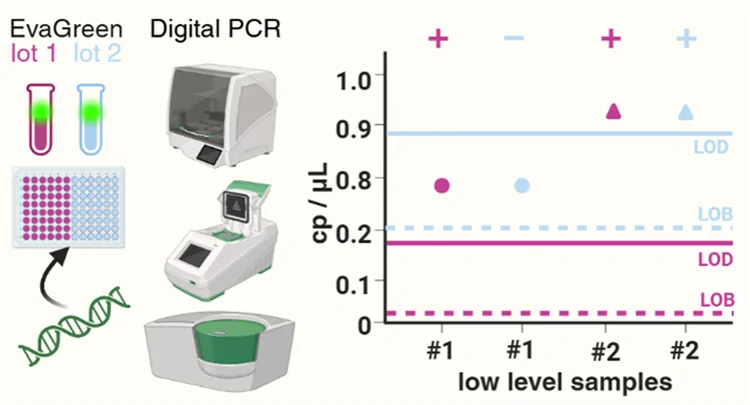

Since its invention in the 1980s,^1–3^ polymerase chain reaction (PCR) has been advanced for many applications, including but not limited to pathogen detection, and laid the stepstone for amplification-based methods like next-generation sequencing.^4^ Traditionally, PCR was run until its endpoint, amplicons were visualized and analyzed by Southern blotting^1^ or gel electrophoresis.^2^ Ensuing development brought methods like limiting dilution PCR^3,5^ as conceptual predecessor for digital (d)­PCR^6^ or quantitative (q)­PCR^7^ to the market.

As compared to conventional endpoint PCR, nested (n)­PCR increases sensitivity and specificity by two subsequent PCR runs. Specificity is then determined for example on a gel.^2^ In qPCR, either DNA-binding fluorescent dyes or fluorescently labeled probes allow the real-time detection of the target sequence.^7,8^ In contrast to endpoint PCR, this allows quantification, obviates laborious post-PCR handling, and simultaneously decreases the contamination risk. Extension to the 96-well format enables automation, high throughput, and short turnaround times.^7^ However, the amplification efficiency in qPCR largely depends on template and primer structure, sample matrix, reagents, and competing reactions, and the target quantification is relative to a standard curve.^9^ In DNA-binding dye-based applications, melt-curve analysis can be used to interpret the assay specificity.^10^ On the other hand, dPCR usually combines cycling to endpoint with the fluorescence read-out concept.^11^ In modern dPCR, a typical DNA-binding dye-based reaction contains template, primers, and the dPCR chemistry (often given summarizing names like “Supermix”), which consists of at least the DNA-binding fluorescent dye, DNA polymerase, dNTPs, and Mg^2+^ ions.^12^ Preamplification, the assembled reaction is dispersed into thousands of either nanosized droplets or solid chambers on microfluidic chips. Conventionally, each partition is cycled until endpoint and analyzed in a binary (digital) manner. Assuming random target distribution,^11^ Poisson statistics are then applied to infer the absolute target concentration based on the cluster of fluorescence-negative droplets.^13^ Partitioning is thought to render dPCR less prone to certain inhibitors and amplification inefficiency, increase sensitivity, precision, and reproducibility, and allow the amplification of minute amounts of target sequence within high concentrations of background DNA.

Regardless of the exact concept, PCR enables the analysis of nucleic acids in concentrations otherwise too low to detect. For applications with an extremely small expected target amount, the validation of the PCR assay’s low range is especially important.^14^ Crucial terms in this context are the limit of blank (LOB), describing the highest signal within blank measurements, limit of detection (LOD), and limit of quantification (LOQ). The Clinical and Laboratory Standards Institute (CLSI) EP17-A guideline^15^ describes the LOD as the assay’s capability to distinguish low concentrations from the assay background, arising from the instrument itself as well as from the sample matrix devoid of target (blank). The LOQ thereafter is the lowest reliably quantifiable concentration, where the measurement uncertainty, expressed as coefficient of variation (CV), is less than or equal to a preset goal.

PCR can improve, simplify, or complement infection diagnostics in cases where culture and serology are laborious or low in specificity,^16–18^ so for example the diagnosis of *Chlamydia pneumoniae* infections. *C. pneumoniae* is an obligate intracellular human respiratory pathogen. Infections are ubiquitous, and a substantial proportion remains asymptomatic.^19–21^ Despite this, *C. pneumoniae* can switch to a persistent state within the host that survives phagocytosis. Persistently infected peripheral blood mononuclear cells (PBMC) disseminate the bacteria to distant body sites, where contributions to chronic inflammatory diseases have been suggested.^22^ Due to the laborious nature of culture methods and the low specificity and sampling time-dependent sensitivity of serological assays, active infection diagnostics have undergone a shift toward nucleic acid amplification tests.^19^ And whereas these are also strongly endorsed by bodies like the U.S Centers for Disease Control and Prevention,^23^ a tool for persistent infections does not exist to date.^24^

The intracellular chlamydial nature implies the coisolation of large amounts of human DNA. These compete in downstream analyses, and the bacterial distribution throughout the body in persistent cases further reduces the detection chances. Given dPCR’s reported advantages for exactly these cases, we hypothesized that dPCR is the method of choice to detect trace amounts of chlamydial DNA within a high human DNA background. We developed, optimized, and validated two dPCR assays, detecting the chlamydial 16S rRNA gene and the *groL* gene, respectively. Obstacles could not be overcome by first-line approaches of method optimization. Instead, we found that intra- and interassay fluctuations, which impacted the LOB and consequently LOD by a factor of ≤ 5, were attributable to batch-to-batch differences in the dPCR chemistry, and that recovery is an inverse function of sample storage time. We concluded that this method needs the establishment of lot-specific LOBs and LODs to allow reliable detection in low-level samples, but even then nPCR prevailed regarding sensitivity.

## Experimental Section

### Cell Culture and Genomic DNA Isolation

*C. pneumoniae* cardiovascular isolate strain CV-6 ^25^ (Paracelsus Medical University, Salzburg, Austria) was propagated in A549 cells as previously described^26^ and genomic (g)­DNA isolated from chlamydial elementary bodies following the GeneJET Genomic DNA Purification Kit (Thermo Scientific, Vilnius, Lithuania) “Gram-Negative Bacteria Genomic DNA Purification Protocol”.

THP-1 cells (ATCC-TIB-202; ATCC, Manassas, VA, USA) were grown in RPMI-1640 medium (Cytiva, Logan, UT, USA) supplemented with a final concentration of 10% heat-inactivated fetal bovine serum (FBS; Biowest, Nuaillé, France), 20 μg/mL gentamicin, and 0.05 mM 2-mercaptoethanol (both Gibco, Grand Island, NY, USA) at 37 °C (5% CO_2_, 95% humidity). Y79 cells (ECACC 86093003) were obtained from Professor Timo Laaksonen (University of Helsinki, Helsinki, Finland) and used directly for gDNA extraction.

Cultured cell gDNA was isolated with the GeneJET kit “Cultured Mammalian Cells Genomic DNA Purification Protocol”. Immediately afterward, its concentration was measured on the NanoDrop One UV–visible spectrophotometer equipped with software v2.6.0.5 (Thermo Scientific, USA) and on a Qubit Flex Fluorometer (Invitrogen, Singapore) using the Qubit 1× dsDNA HS Assay Kit (Invitrogen, Eugene, OR, USA). DNA samples were stored at −20 °C directly after concentration estimation.

### Plasmid Construction

The 16S rRNA and *groL* inserts were amplified from CV-6 gDNA with 16S rRNA, *groL*_2, and *groL*_3 Cloning primers (Metabion, Planegg, Germany; SI Tab. 6), digested with *Bam*HI- and *Hin*dIII-HF (16S rRNA insert) or *Hin*dIII- and *Sbf*I-HF (*groL* inserts), and cloned into pUC19. High Efficiency NEB 5-α Competent *E. coli* (all New England Biolabs, Ipswich, MA, USA) were heat shock-transformed, plasmids isolated with the NucleoSpin Plasmid Easy Pure Kit (Macherey-Nagel, Düren, Germany), and termed 16S-pUC19 (2851 bp), *groL*-pUC19_2 (3314 bp), and *groL*-pUC19_3 (3346 bp). Quality control ensued via Sanger sequencing (DNA Sequencing and Genomics Laboratory, University of Helsinki, Helsinki, Finland) and was interpreted in SnapGene v8.0.1 (SnapGene, Boston, MA, USA). Plasmids were further separated on an agarose gel from coisolated *E. coli* gDNA. The remaining gDNA amount was assessed by qPCR as described below, whereby the *E. coli* genome size and molar mass per base pair were estimated with 4.7 × 10^6^ bp/genome and 650 (g/mol)/bp, respectively. The *E. coli* mass-specific gDNA amount was calculated following eq 1, where *N*_*A*_ denotes the Avogadro constant. The qPCR standard curve was based on these calculations and the resulting copy numbers normalized to the mass of plasmid template.

1 $$\mathrm{cp}/\mathrm{ng}\;\mathrm{DNA}=\frac{N_{A}}{3.06\times 10^{18}\;\mathrm{ng}/\mathrm{mol}}$$

### Digital PCR

The optimized dPCR protocol was as follows (changes indicated in the Results section): Digital PCR reactions were prepared at room temperature (RT) in 96-Well Semi-Skirted ddPCR plates in a total volume of 22 μL, of which 20 μL contained 1× QX200 ddPCR EvaGreen Supermix (further referred to as Supermix; both Bio-Rad Laboratories, USA), 200 nM of each forward and reverse primer (*CPN*-16S or *CPN-groL*; SI Tab. 6), 5 U *Sca*I-HF, and sample. Samples were 1–10,000 CV-6 genome copies, sole plasmid in different concentrations, or plasmid spiked into 125 ng THP-1 gDNA (matrix). Reactions were filled up with nuclease-free water (NFW; Thermo Scientific, Vilnius, Lithuania) and subjected to partitioning in the QX200 Automated Droplet Generator (Bio-Rad Laboratories, USA). Used Supermix lots are listed in Table 5. Droplet generation happened in DG32 Automated Droplet Generator Cartridges (Bio-Rad Laboratories, Germany) utilizing QX200 Droplet Generation Oil for EvaGreen (Bio-Rad Laboratories, USA) after 15 min *Sca*I-digest at RT. The individual droplet volume was 0.85 nL, resulting in 40 μL postpartitioning volume. PCR plates were sealed with Pierceable Foil Heat Seals (Bio-Rad Laboratories, UK) and thermocycled on a Veriti 96-Well Thermal Cycler (Applied Biosystems, Singapore). The manufacturer’s default cycling program was used if not further specified, and multiple protocols (Table 1) were compared. All runs included at least one positive (plasmid spiked into matrix) and one negative control (matrix only) as well as a nontemplate control (NTC), in which NFW substituted DNA. Droplets were evaluated by a QX200 Droplet Reader (Bio-Rad Laboratories, USA) and data analyzed using QuantaSoft software v1.7.4. (Bio-Rad Laboratories, Hercules, CA, USA). A reaction passed quality control if the number of accepted droplets exceeded 10,000. Partition classification (thresholding) was conducted manually whereby the threshold was set right between the negative and positive cluster based on the positive control. The fluorescence amplitude was expressed as relative fluorescence units (RFU).

**Table 1:** Digital PCR Thermocycling Profiles^a^

Protocol	Profile
Default	95.0 °C −5 min, 40× (95.0 °C – 30 s, 60.0 °C – 1 min), 4.0 °C – 5 min, 90.0 °C – 5 min, 4.0 °C – infinite
”A”	95.0 °C – 5 min, 40× (95.0 °C – 30 s, 65.0 °C – 1 min), 4.0 °C – 5 min, 90.0 °C – 5 min, 4.0 °C – infinite
”B”	95.0 °C – 5 min, 40× (95.0 °C – 30 s, 65.0 °C – 2 min), 4.0 °C – 5 min, 90.0 °C – 5 min, 4.0 °C – infinite
”C”	95.0 °C – 5 min, 40× (95.0 °C – 30 s, 60.0 °C – 1 min), 4.0 °C – 5 min, 90.0 °C – 5 min, 4.0 °C – infinite
“D”	95.0 °C – 5 min, 40× (95.0 °C – 1 min, 65.0 °C – 2 min), 4.0 °C – 5 min, 90.0 °C – 5 min, 4.0 °C – infinite

a The ramp rate in all steps was 50% except for 25% in profile “C”.

#### Restriction Digest Verification and Recovery Along Sample Storage Time

Digest of 137.5 ng 16S-pUC19 or THP-1 cell gDNA with 5.5 U of *Hin*dIII-, *Bam*HI-, *Sbf*I-, or *Sca*I-HF was set up in 1× Supermix and filled up to 22 μL with NFW. A digest control in 1× rCutSmart Buffer (New England Biolabs), and a nondigested control in NFW were included for each DNA source and the reactions incubated for 15 min either at RT or 37 °C. The *groL*-plasmids were set up for *Sca*I-digest confirmation at RT only. Cleavage was evaluated on an agarose gel.

For the time-dependent study, the concentration of a *groL*-pUC19_3 plasmid sample was estimated fluorometrically. A stock solution of 1174 copies (cp) per microliter was created and stored in single-use aliquots at −20 °C. Fourteen dPCRs were conducted over a period of 46 days with two runs on Day 0, 4, 23, and 46. Each PCR used an aliquot from which an input of 1000 cp/well was obtained and freshly spiked into the matrix.

#### Limit of Blank

LOB estimations followed the Clinical and Laboratory Standards Institute (CLSI) EP17-A guideline:^15^ For the assay detecting the chlamydial 16S rRNA (further referred to as 16S-assay), 63 technical matrix replicates (blanks) were subjected to dPCR. For each *groL*-assay the replicate number was 62. The LOB was estimated nonparametrically as the 95th percentile of ranked measurements according to eq 2, where *n*_*B*_ denotes the number of technical replicates. Linear interpolation for noninteger ranks corresponding to the 95th percentile was carried out according to eq 3, where *r*_*low*_ and *r*_*high*_ denote the measured concentration corresponding to the rank below and above the noninteger, respectively, and *f* the rank’s decimal part. All calculations are given in Supporting Information (SI Tab. 1).

2 $$95th\;percentile=\left\lceil n_{B}\times \left(\frac{95}{100}\right)+0.5\right\rceil$$

3 $$\mathrm{LOB}=r_{\mathrm{low}}+f\times \left(r_{\mathrm{high}}-r_{\mathrm{low}}\right)$$

#### Preparation of Low-Concentration Samples

Based around the LOBs, low-concentration samples (LCS) for LOD estimation were created. At equidistant concentrations, each LCS was prepared as a master mix and aliquoted for each PCR run to ensure equal pipetting errors and freeze–thaw conditions. Plasmid 16S-pUC19 was used for the 16S-assay, *groL*-pUC19_2 for the *groL*-assay_1, and *groL*-pUC19_3 for the *groL*-assay_2. The plasmid sizes and the molar mass/bp were applied to obtain the mass-specific amount of plasmid in [cp/ng] as described in eq[Disp-formula eq1]. Each technical LCS replicate was constituted so that a 20 μL-PCR reaction contained matrix and plasmid at the required concentrations in [cp/μL] (Table 2). A representative calculation can be found from SI “Example calculations for low-concentration samples (LCS) for the *groL*-assay_1”.

**Table 2:** Constitution of Low-concentration Samples For Limit of Detection Specification^a^

	# Different Concentrations	Estimated cp/μL	Estimated cp/well
16S-assay	4 (dPCR)	0.27–1.08	5.40–21.6
*groL*-assay_1	7 (d-, n-, qPCR)	0.06–0.24	1.20–4.80
*groL*-assay_2	4 (d-, qPCR)	0.42–1.05	8.40–21.0
	9 (nPCR)	0.01–1.05	0.20–21.0

a Concentrations denote the final plasmid cp/μL-PCR reaction or 20 μL-PCR well (in 125 ng/well THP-1 cell gDNA), whereby the prepartitioning PCR volume was 22 μL/well. **Abbreviations:** dPCR digital PCR; nPCR nested PCR; qPCR quantitative PCR.

#### Limit of Detection

LOD estimation followed the CLSI EP17-A guideline:^15^ LCS in the concentration range stated in Table 2 were subjected to dPCR in 3–4 technical replicates per run, whereby the total number of runs was four per assay on 2–4 different days. This resulted in 15 technical replicates per LCS (*N*_*per group*_) with 60 total measurements (*N*_*s*_) for the 16S-assay and the *groL*-assay_2. For the *groL*-assay_1, *N*_*per group*_ was 16 and *N*_*s*_ 112. The parametric LOD was estimated according to eqs 4–5, where *c*_β_ is derived from the 95th percentile of a Gaussian distribution and the correction factor, and *SD*_*S*_ is the pooled standard deviation. The total degrees of freedom (*f*_*tot*_) are calculated as (*N*_*s*_ – *K*), where *K* is the number of different LCS concentrations (*n* = 4 for the 16S- and *groL*-assay_2, *n* = 7 for the *groL*-assay_1).

4 $$\mathrm{LOD}=\mathrm{LOB}+c_{\beta }\times {\mathrm{SD}}_{S}$$

5 $$c_{\beta }=1.645/\left[1-1/\left(4\times f_{\mathrm{tot}}\right)\right]$$

For obtaining *SD*_*S*_, the replicate measurements in [cp/μL] were grouped by the estimated LCS concentration. To obtain the weighted variances σ_*w*_^2^, the variance σ^2^ of each group was multiplied by the degrees of freedom of each group *f*. The latter were calculated as the number of technical replicates within each group minus 1. The sum of the weighted variances ∑σ_*w*_^2^ was calculated. To obtain the pooled variance σ_*p*_^2^, ∑σ_*w*_^2^ was divided by *f*_*tot*_. Subsequently, *SD*_*S*_ was calculated by taking the square root of the pooled variance. Each assay’s calculations are given in SI Tab. 2–4.

#### Limit of Quantification

The LOQ was calculated according to the CLSI EP17-A guideline,^15^ and calculations were based on the LCS measurements. A proposed CV ≤ 25% for microbial DNA detection by qPCR^27^ was used as cutoff. The lowest estimated concentration among the LCS that was >LOD with a CV ≤ 25%, provided that all higher LCS concentrations also remained below this limit, was defined as the LOQ. Each assay’s calculations are given in SI Tab. 2–4.

#### Linearity, Precision, and Trueness

According to the International Organization for Standardization (ISO) 20395:2019 standard,^11^ linearity, intrarun variation/relative repeatability as a measure of method repeatability, relative run-to-run variation as a measure of intermediate precision, and trueness expressed as relative bias and recovery were determined. Serial dilutions of *groL*-pUC19_3 (0.1–1,000,000 cp/well) in matrix were constructed as master mix and frozen as single-use aliquots at −20 °C. On three different days, technical triplicates and an NTC were subjected to dPCR, resulting in 8–9 measurements per concentration (Supermix lot 64731978). Data were analyzed based on the ISO 20395:2019 standard^11^ and Gleerup et al.^28^ Raw measurement data, formulas, and calculations are provided in SI Tab. 5.

#### Comparison of Amplicon Sizes

Reactions for dPCR were prepared as described above but 10 ng gDNA from *C. pneumoniae*-infected PBMCs per 20 μL-reaction served as template. Seven technical replicates were amplified with the *CPN*-16S primers, another set with the *CPN-groL* primers, and RFU, detected concentrations, and accepted droplet counts compared between the assays.

### Quantitative PCR

The qPCR reactions were carried out as described^29^ with the following modifications: LCS or 20 ng *E. coli* gDNA were amplified in technical triplicates with 200 nM of either *CPN-groL* or *EC* primers (SI Tab. 6) in reactions filled up to 20 μL with NFW. The concentrations of unknown samples were inferred from a five-point standard curve and specificity was determined by melt-curve comparison of unknown samples with standards. During sensitivity comparison of the different PCRs, an LCS was reliably detected when all replicates showed the specific melt-curve profile.

### Nested PCR and Agarose Gel Electrophoresis

Each outer PCR reaction, including positive controls and NTCs, was prepared on ice in thin-walled 0.2 mL tubes (Thermo Scientific, China) and consisted of 1× DreamTaq Buffer, 0.2 mM dNTP Mix, 1.25 U DreamTaq DNA Polymerase (all Thermo Scientific, Vilnius, Lithuania), 200 nM primers (SI Tab. 6), and LCS in technical triplicate. The primers were pUC19 Seq (*groL*-assay_1) and *CPN-groL*_out primers (*groL*-assay_2) and the reactions filled up to 20 μL with NFW. Thermocycling was conducted on a T100 Thermal Cycler (Bio-Rad, Singapore): 95.0 °C – 3 min, 30× (95.0 °C – 1 min, AT – 1 min, 72.0 °C – 1 min), 72.0 °C – 7 min, 12.0 °C – infinite, where AT denotes the annealing temperature (SI Tab. 6). Five microliters outer PCR product were transferred to the inner PCR reaction, using the same chemistry and thermocycling but *CPN-groL*_in primers in both assays. GeneRuler 100 bp DNA Ladder, GeneRuler 100 bp Plus DNA Ladder, or GeneRuler 1 kb DNA Ladder (all Thermo Scientific, Vilnius, Lithuania) served as size markers. Imaging was conducted on a ChemiDoc MP Imaging System (Bio-Rad Laboratories, USA). During sensitivity comparison of the different PCRs, an LCS was called reliably detected if all technical replicates showed a 440 bp-band for the inner amplicon.

### Statistical Analysis

Statistical analysis was carried out using Statistics Kingdom (Statistics Kingdom, Melbourne, Australia; www.statskingdom.com). Linearity was assessed in IBM SPSS Statistics v32.0.0.0 (IBM Corp., Armonk, NY, USA). The α-level was 5% and all given *p*-values are two-tailed. Normality and homoscedasticity assumptions were tested with the Shapiro-Wilk test and Levene’s test, respectively. Comparison of two groups with violated assumptions was performed using Wilcoxon Signed-Rank or Mann–Whitney U tests, for more than two groups Friedman tests with Nemenyi posthoc analysis were conducted. All data are presented as mean ± standard deviation (SD). Significances are indicated by ns (*p* > 0.05, nonsignificant); * (*p* < 0.05); ** (*p* < 0.01); *** (*p* < 0.001). Graphs were created in Microsoft 365 Excel v2508 (Microsoft Corporation, Redmond, WA, USA).

## Results

Optimization needs during the dPCR assay development could be narrowed down rapidly to two distinct factors: (i) the occurrence of FPDs and (ii) the inconsistent but mainly decreasing recovery of estimated DNA concentrations between runs. All optimization steps were tailored to overcome these deficiencies to develop a robust and reliable dPCR assay.

### 16S-Assay Optimization

#### Annealing Temperature and Primer Concentration

The initial assay optimization was attempted with *C. pneumoniae* CV-6 gDNA. Based on the spectrophotometrically measured concentration of the CV-6 gDNA stock, an estimated number of 10,000; 1000; 100; and 10 cp/well (*n* = 1 per concentration and temperature), two technical replicates á 40 ng/well THP-1 cell gDNA as a negative control, and one NTC were subjected to dPCR following the default thermocycling profile but with annealing/extension temperatures from 55–65 °C (2 °C increments). All temperatures revealed similar detection capability, but detected concentrations were only between 7.4–22% of the estimated DNA input. The baseline fluorescence of negative droplets increased directly proportionally with the temperature from 4889 ± 396 RFU at 55 °C to 6037 ± 283 RFU at 65 °C (Friedman test *p* < 0.001***, *n* = 6; Figure 1F). Nemenyi posthoc analysis revealed significant differences between the temperatures 63 and 65, 57 and 65 °C (both adj. *p* = 0.003**), as well as 55 and 65 °C (adj. *p* < 0.001***), but this did not affect the separation of negative and positive clusters (Figure 1A–D). All annealing/extension temperatures except 65 °C yielded 1–4 FPDs in the negative controls (Figure 1E). With 23,538 ± 3072 RFU (*n* = 5), FPDs showed a decreased mean fluorescence amplitude compared to true positives with 29,889 ± 389 RFU (*n* = 19; Mann–Whitney U test *p* < 0.001***). However, the FPD count exceeded the number of positive droplets for the lowest gDNA input and therefore hampered interpretation. Only at 65 °C, 10 cp/well could be confidently defined as positive. As this temperature showed a good cluster separation, comparable detected concentrations, detection even in the sample with the lowest concentration, and the highest specificity, it was chosen for all subsequent assays. Increasing the primer concentrations from 200 to 400 and 600 nM only resulted in the elevation of the baseline fluorescence from 5297 ± 569 to 8181 ± 149 to 10,231 ± 194 RFU, respectively (Friedman test *p* < 0.001*** with differences between 200 and 600 nM as found by Nemenyi posthoc analysis adj. *p* < 0.001***; *n* = 7 per concentration), while leaving recovery and droplet separation unaltered (data not shown). The recovery of the estimated input across all primer and sample concentrations was 7.6–13.8% (with one exception of 30% for the recovery of 10 chlamydial genomes with 600 nM primers). Therefore, we used the 200 nM primer concentration in all subsequent experiments.

**Figure 1:**
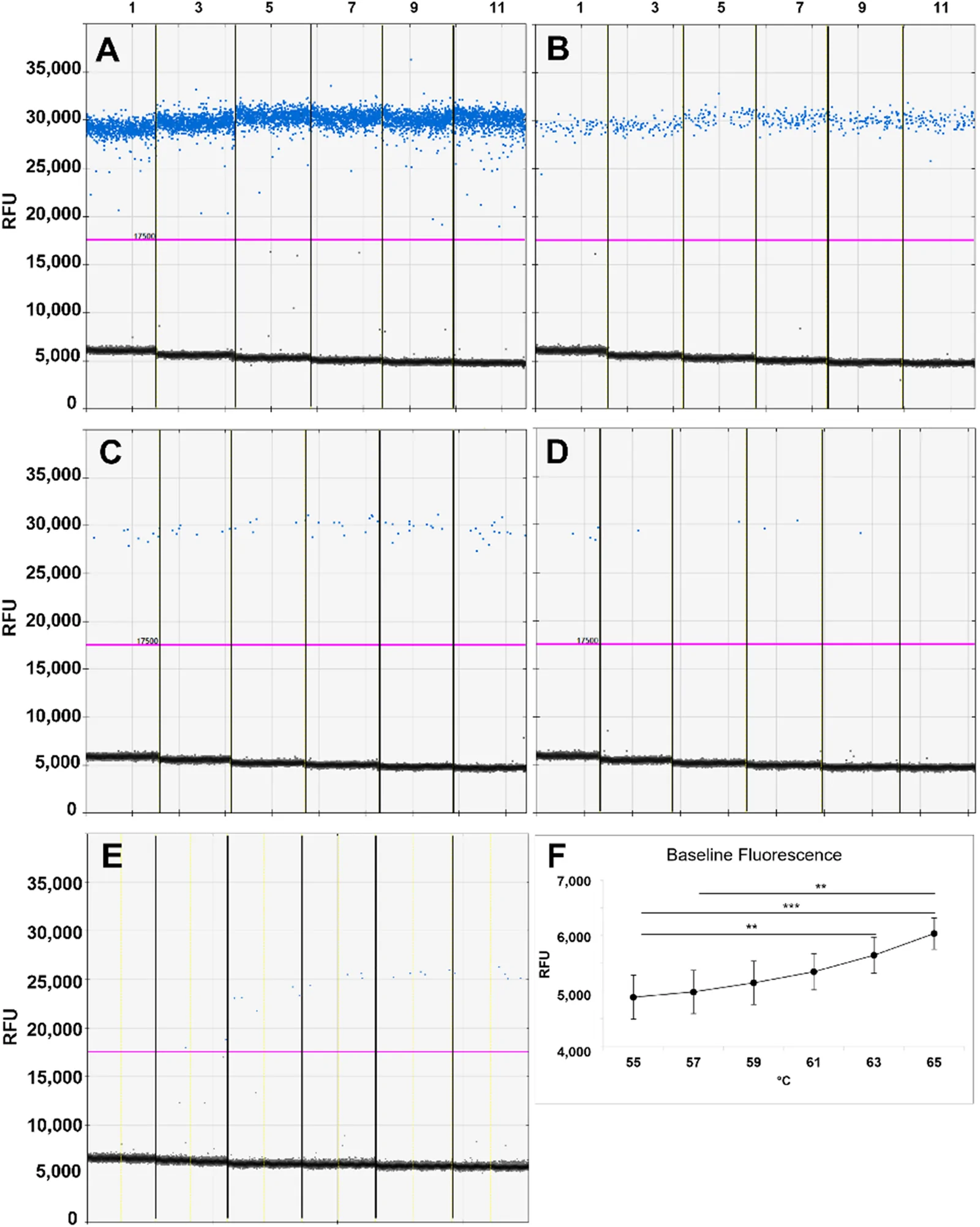
One-D amplification plots of a temperature gradient PCR on the QX200 Droplet Digital PCR System with (A) 10,000; (B) 1000; (C) 100; (D) 10 chlamydial genomes per 20 μL-well (*n* = 1 per concentration and temperature), and (E) 40 ng/well THP-1 gDNA as negative control (technical duplicates). Columns 1/3/5/7/9/11: 65–55 °C. All droplets >17,500 RFU-threshold are positive. (F) Negative droplets fluorescence based on temperature and irrespective of concentration. Data are presented as mean ± standard deviation (*n* = 6 per temperature). Friedman test with Nemenyi posthoc test (*p* < 0.001***) reveals differences between 55 and 63, 57 and 65 °C (both adj. *p* = 0.003**), as well as 55 and 65 °C (adj. *p* < 0.001***).

#### Plasmid Construction

We hypothesized that the chlamydial gDNA stock contained an unknown amount of coisolated human gDNA, thus resulting in incorrect input calculations and low recoveries. Therefore, 16S-pUC19 was constructed and coisolated *E. coli* gDNA reduced by gel separation, which yielded 115 ± 12 *E. coli* genomes per nanogram plasmid in qPCR. For a theoretical dPCR input of 10,000 plasmids, 31 fg plasmid DNA would be required, which contain on average 3.5 × 10^–3^ ± 3.7 × 10^–4^ *E. coli* genomes. *GroL*-plasmids behaved similarly. Furthermore, LCS contained plasmid only at the attogram level, hence the contribution of *E. coli* gDNA to the recovery was deemed negligible.

#### Thermocycling Profiles

Ten thousand copies of spectrophotometrically measured 16S-pUC19 were *Hin*dIII-digested within the dPCR reaction (profile “A”). For sole and spiked-in plasmid the recoveries were 48.1 ± 1.0% and 49.5 ± 0.4%, respectively (*n* = 2 technical replicates, each). The FPD count was 1 in the negative control, but the 1-D amplitude plots showed many droplets with intermediate fluorescence, a phenomenon termed “rain”,^11^ which complicated partition classification as intermediate droplets could not be confidently interpreted. With ∼50% recovery, the use of plasmid seemed to allow a better estimation of the DNA input, however, it did not convey sufficient accuracy to the assay, and the “rain” was troublesome. We attempted to increase droplet separation and recovery by varying the thermocycling profiles as parameters like denaturation or annealing/extension time could facilitate droplets to exceed the threshold.^12^ Table 3 lists all applied profiles and the observed recoveries.

**Table 3:** Recovery in Digital PCR across Different Thermocycling Profiles^a^

Thermocycling Profile	Input [cp/well] (n)	Recovery [%]	# FPD	“Rain”
“A”	10,000 (2)	48.8 ± 1.0	1	yes
“B”	10,000 (2)	59.5 ± 3.4	1	yes
”D”	10,000 (1)	41.6 ± nA	1	yes
”B”, 50 cycles	10,000 (2)	27.9 ± 0.3	0	improved
”A”, 50 cycles	100–10,000 (6)	23.7 ± 1.8	1–2	10,000 cp/well
”B”	100–10,000 (6)	23.4 ± 4.7	0–2	10,000 cp/well
“A”, in-dPCR *Sca*I-digest	100–10,000 (5)	24.8 ± 1.3	0	10,000 cp/well
“A”, pre-dPCR*Sca*I-digest	100–10,000 (6)	14.3 ± 2.8	9	improved

a Digital PCRs were conducted with different input concentrations (100; 1000; and 10,000 cp/well) of either 16S-pUC19 plasmid or a combination of 125 ng THP-1 gDNA with spiked-in plasmid (*n* = 2 technical replicates per concentration and sample constitution except for the combination with *Sca*I-digest, where *n* = 1). Data are presented as mean ± standard deviation of the same concentrations irrespective of the sample composition. Note that there was only 1 week difference between runs with profile “B”. **Abbreviations:** FPD false positive droplets; nA not available; “rain” droplets of intermediate fluorescence.

Although the recovery fluctuated and mainly decreased, it was mostly in the same range across all dilutions and independent from the sample constitution. This indicated an absent matrix effect. The restriction enzyme substitution within the dPCR reaction resulted in improved “rain” and absent FPDs. When the *Sca*I-digest was conducted pre-dPCR, diluted and added to the dPCR, the “rain” also improved for an input of 10,000 cp/well. Simultaneously, the recovery dropped to 14.3 ± 2.8% (*n* = 6) and the FPD count rose to 9. This raised the suspicion that *Hin*dIII- and *Sca*I-HF have different compatibilities with the Supermix.

#### Restriction Digest Verification

For DNA digest, the initial dPCRs used 5.5 U *Hin*dIII-HF directly added to the 22 μL-reaction. A verification of this digest in Supermix resulted in 1.5–2 kb-bands for supercoiled plasmid and a high molecular weight band for gDNA on an agarose gel, indicating insufficient *Hin*dIII-digest (RT, 15 min). Digest only occurred at 37 °C (data not shown). The same was observed for *Bam*HI-HF, recommended as an alternative in personal communication with Bio-Rad Laboratories. Only *Sca*I-HF showed sufficient digest, indicated by a 2.5–3 kb-band for 16S-pUC19 and a smear for gDNA (Figure 2A,B). The same held true for both *groL*-plasmids (Figure 2C). As the plasmid concentrations used across all dPCR reactions remained far below the ones used for the gel-based verification to allow visualization, the digest was considered sufficient although a fraction remained in its supercoiled (1.5–2 kb) or open circular monomer (>2.5–3 kb) conformation. *Sca*I-HF was subsequently used in all dPCRs reactions. Nevertheless, the substitution did not improve the recovery.

**Figure 2:**
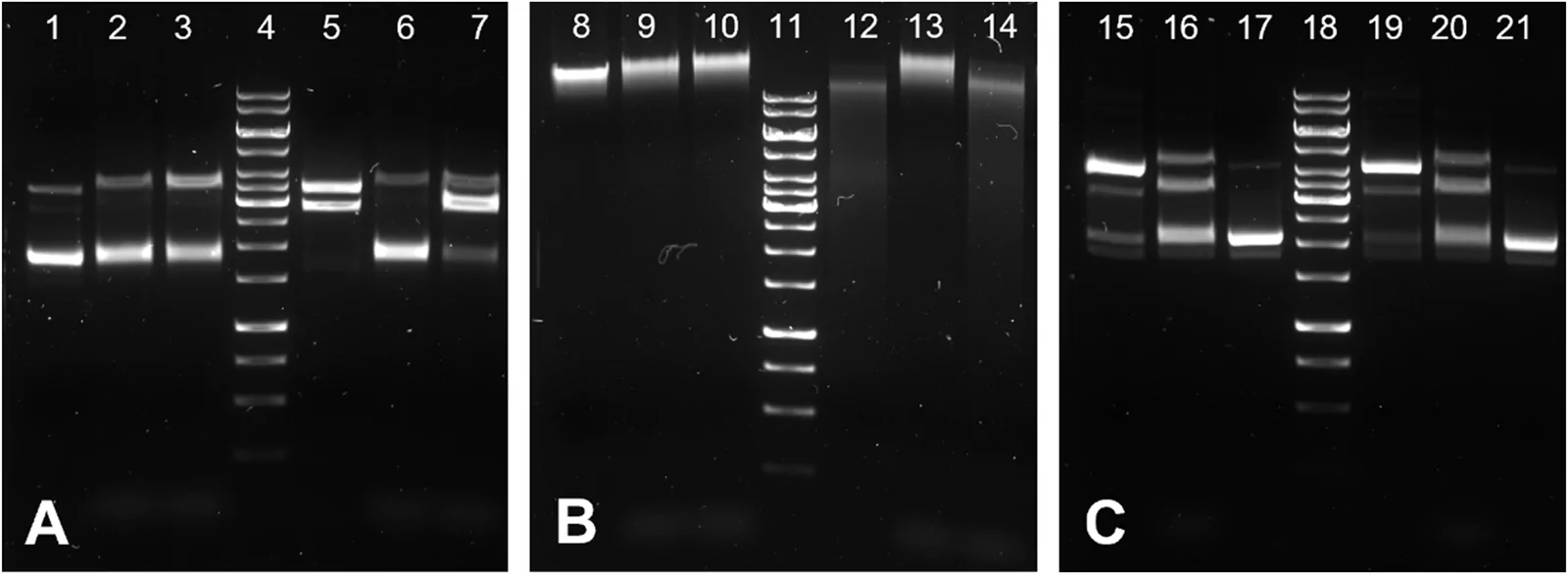
Digest evaluation of (A) 16S-pUC19, (B) THP-1 gDNA, and (C) *groL*-pUC19_2 and _3. Lane (L)­1: nondigested 16S-pUC19 control; L2: nondigest by *Bam*HI-HF; L3: nondigest by *Hin*dIII-HF; L4: 1 kb ladder; L5: 16S-pUC19 digest control in rCutSmart Buffer with *Hin*dIII-HF; L6: nondigest by *Sbf*I-HF (noncutter on all plasmids); L7: *Sca*I-digest. Restriction enzymes in L8–14 are distributed as in Figure 2A but gDNA stems from THP-1 cells. L15: *groL*-pUC19_2 digest control in rCutSmart Buffer with *Hin*dIII-HF; L16: sufficient *Sca*I-digest; L17: nondigested *groL*-pUC19_2 control; L18: 1 kb ladder. Restriction enzymes in L19–21 are distributed as in L15–17 but shown for *groL*-pUC19_3.

#### Spectrophotometric versus Fluorometric Determination of DNA Concentrations

We next compared the DNA concentration measurements between the NanoDrop One UV–visible spectrophotometer and the Qubit Flex Fluorometer to evaluate if the measurement method might be responsible for the poor recovery. For spectrophotometry, between 1.7- and 4.1-times higher plasmid concentrations were observed (Wilcoxon Signed-Rank test *p* = 0.004**, *n* = 9; Figure 3A). This effect was inconsistent for gDNA: In five of nine samples, spectrophotometry showed 1.1- to 1.4-times higher values than fluorometry, but in the remaining four samples 1.1- to 1.5-fold lower ones (Wilcoxon Signed-Rank test *p* = 0.910 ns, *n* = 9; Figure 3B). Based on these observations, the prestorage DNA quantitation method was changed to fluorometry, but the recovery kept decreasing during the subsequent dPCR runs.

**Figure 3:**
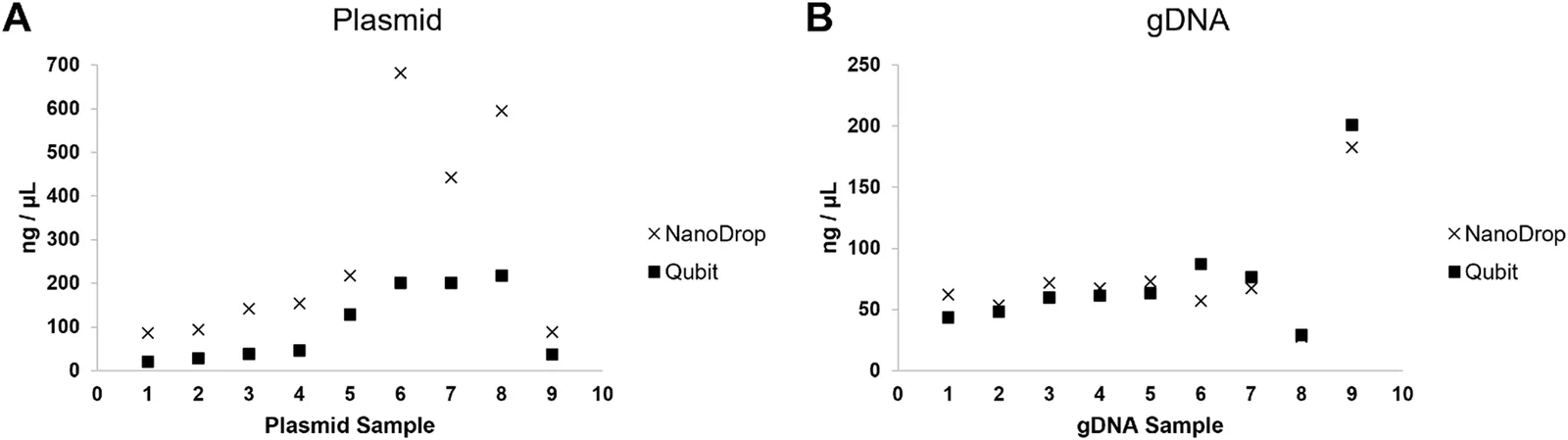
NanoDrop One versus Qubit Flex Fluorometer measurements of (A) nine plasmid and (B) nine gDNA samples. Fluorometric measurements utilized the Qubit 1× dsDNA HS Assay Kit. Data are presented as single measurement points. Wilcoxon Signed-Rank test *p* = 0.004** for comparison of measurement methods for plasmids and *p* = 0.910 (ns) for gDNA samples.

#### Plasmid Recovery and Sample Storage Time

Neither the measurement discrepancy nor the choice of restriction enzyme, the coisolation of gDNA, or the thermocycling profiles could explain the primarily decreasing recovery until we discovered that freshly isolated plasmid yielded better recovery. For an input range of 1, 10, and 100 cp/well, the recovery was 72–220% (*n* = 2 independent runs with profile “A” on consecutive days with new dilutions of recently isolated 16S-pUC19 prepared from single-use aliquots). In one of four wells containing 1 cp/well the detection failed. This observation provided an interim explanation for the decreased recovery in each run, and the 16S-assay specification was continued.

Later, the hypothesis of an inverse relationship between storage time and recovery was experimentally confirmed over 46 days (Figure 4). Whereas the recovery was 116.8 ± 3.7% on the stock preparation day, it was 121.4 ± 23.2% on Day 4 but decreased to 67.6% after 1 week of storage. Except on Day 4, the intraday variability was small as implied by the SD: 16.7 ± 1.3% (Day 23), and 3.9 ± 1.0% (Day 46). This indicated reliable instrument performance but a decreased recovery over the storage time at −20 °C. Thus, all subsequent dPCRs were performed within 4 days of plasmid isolation or after fluorometrically re-estimating the DNA concentration.

**Figure 4:**
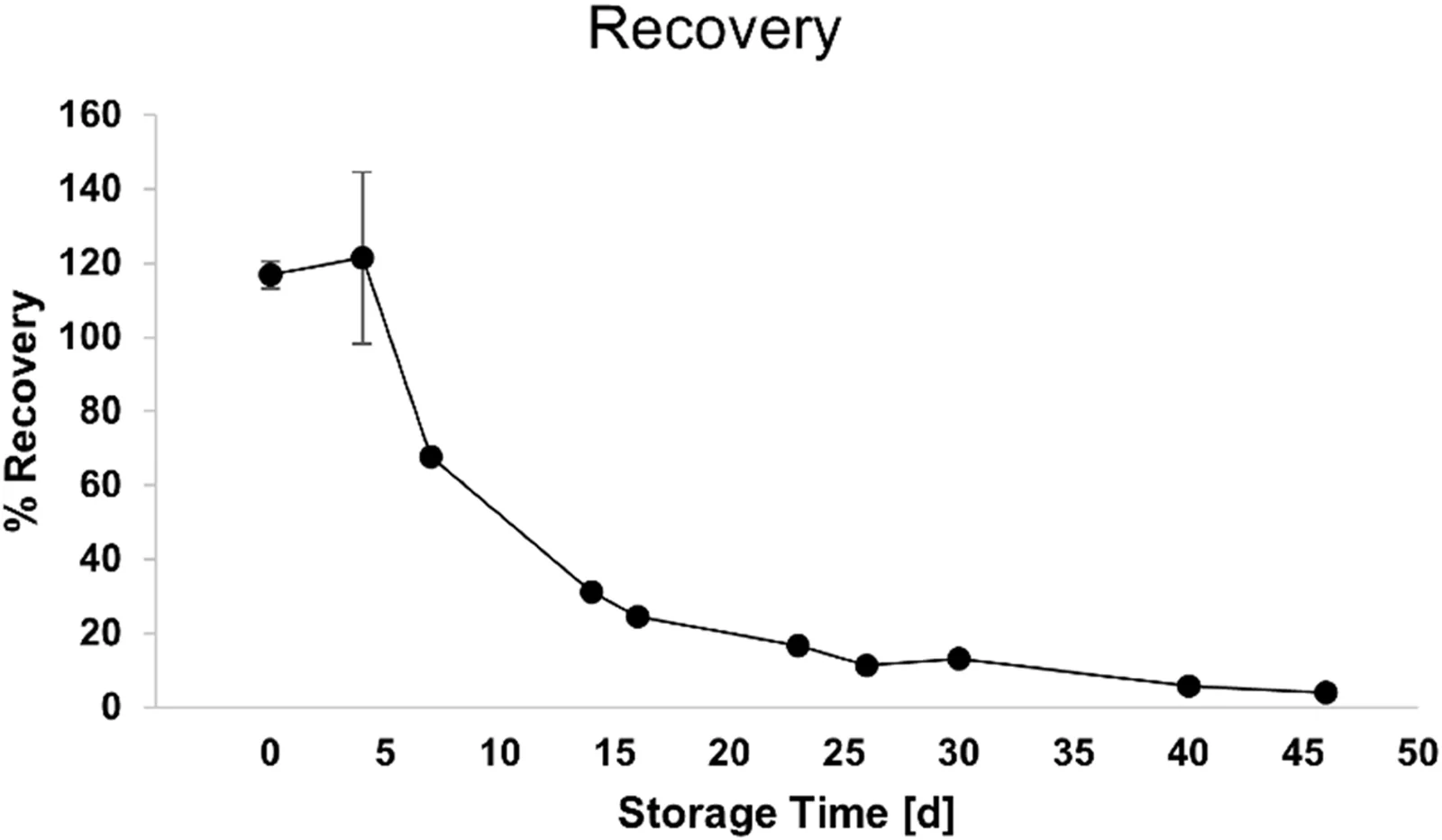
Sample storage negatively impacts recovery in digital (d)­PCR over time. Single-use aliquots of *groL*-pUC19_3 were created, stored at −20 °C, and over 46 days subjected to dPCR á 1000 cp/well spiked into 125 ng/well THP-1 gDNA. Measurements represent single dPCR runs for each day except for Day 0, 4, 23, and 46 (*n* = 2, expressed as mean ± standard deviation).

### 16S-Assay

#### Target Abundance

*C. pneumoniae* detection from blood is hampered by the relatively low abundance of persistent bacteria, especially in asymptomatic individuals,^30–32^ thus, the developed dPCR assay was first maximized for its DNA input. Upon subjecting 16S-pUC19 spiked into 250 ng/well matrix to profile “A”, the negative droplets baseline and FPD count rose to 12,538 ± 492 RFU and 20, respectively, impacting partition classification. Consequently, 125 ng/well as input was chosen. Independent from the assay performance and assuming a Poisson distribution of the target molecules, this theoretically provides ∼95% probability of sampling at least one target molecule present at a frequency of 0.008% (equivalent to 1 chlamydial genome in ∼6 × 10^3^ cells or ∼42 ng human gDNA).

#### Limit of Blank, Limit of Detection, and Limit of Quantification

The first attempt to establish the 16S-assay LOB was conducted with Supermix lot 64639593 (profile “A”). The LOB was 0.18 cp/μL, corresponding to 3.60 cp/well. Thereafter, it was advised to use 60 °C for annealing/extension unless the temperature impacted cluster separation (Bio-Rad Laboratories, personal communication). Since we had established that 60 as well as 65 °C achieved equally good separation (Figure 1), we established an LOB following the default thermocycling profile (Supermix lot 64661060). This LOB was 0.28 cp/μL (Figure 7A), translating to 5.60 cp/well. Based on the measurement of LCS, the LOD was 0.74 cp/μL (Figure 7B), equaling 14.8 cp/well. The LOQ was 0.81 cp/μL or 16.2 cp/well. However, LOB comparison made us question if the default profile was indeed the better choice, since profile “A” showed fewer FPDs. We decided to repeat the LOB estimation with profile “A” (Supermix batch 64661060). This LOB was 0.35 cp/μL, equaling 7.00 cp/well, and was therefore 1.9-fold higher than initially estimated. These nonreproducible results made us suspect a problem with the blanks.

#### Incompatibility of the GeneJET Kit

Y79 cells, obtained from a department not engaged in bacterial work, served to exclude a possible latent *C. pneumoniae* infection in our THP-1 cells. All steps were carried out in the same department and applied single-use, sterile-packed consumables only, which were additional wiped with a Surface Decontaminant. Genomic DNA was extracted with a previously unopened GeneJET kit and subjected in technical replicates to the 16S-assay dPCR. The FPDs persisted: In one of eight technical replicates, 1 FPD could be observed, and endpoint PCR showed a faint band at the same size as the positive control (189 bp, Figure 5A). Four of five THP-1 cell gDNA samples, isolated with another previously unopened GeneJET kit, showed a similar band and three of them additional unspecific bands (Figure 5B). To investigate at which stage the foreign DNA was introduced, we obtained three extraction blanks (sterile phosphate-buffered saline instead of cells as starting material) from the latter kit. Endpoint PCR with these and the *CPN*-16S primers, showed an 189 bp-band in all blanks. In one replicate, two unspecific 200–350 bp-bands occurred (data not shown). To evaluate if the DNA was introduced by *Chlamydia* spp., which are known to infect cattle^33^ and might therefore contaminate FBS, gDNA was isolated from three replicates of each: FBS, culture supernatant, and supplemented THP-1 cell medium. The 189 bp-band was observed in all extracted samples but not the crude, starting material (data not shown). We concluded that the DNA was not cell culture-derived, and since all NTCs were negative as well, contamination of PCR reagents was ruled out too. Three 189 bp-bands from THP-1 cell gDNA (Figure 5B) were examined by Sanger sequencing using the PCR primers. Only 99 bp were visible in the chromatograms and 100% identical with the *C. pneumoniae* 16S rRNA gene when interpreted with NCBI Standard Nucleotide BLAST. One sample additionally showed 86% sequence identity with the human *ATP8A2* gene. Since the contamination source remained unidentified, we redesigned the assay. Samples used in the *groL*-assays were evaluated in endpoint PCR prior to specification to ensure absence of unspecific amplification with the *CPN-groL* primers (representative examples in Figure 5D).

**Figure 5:**
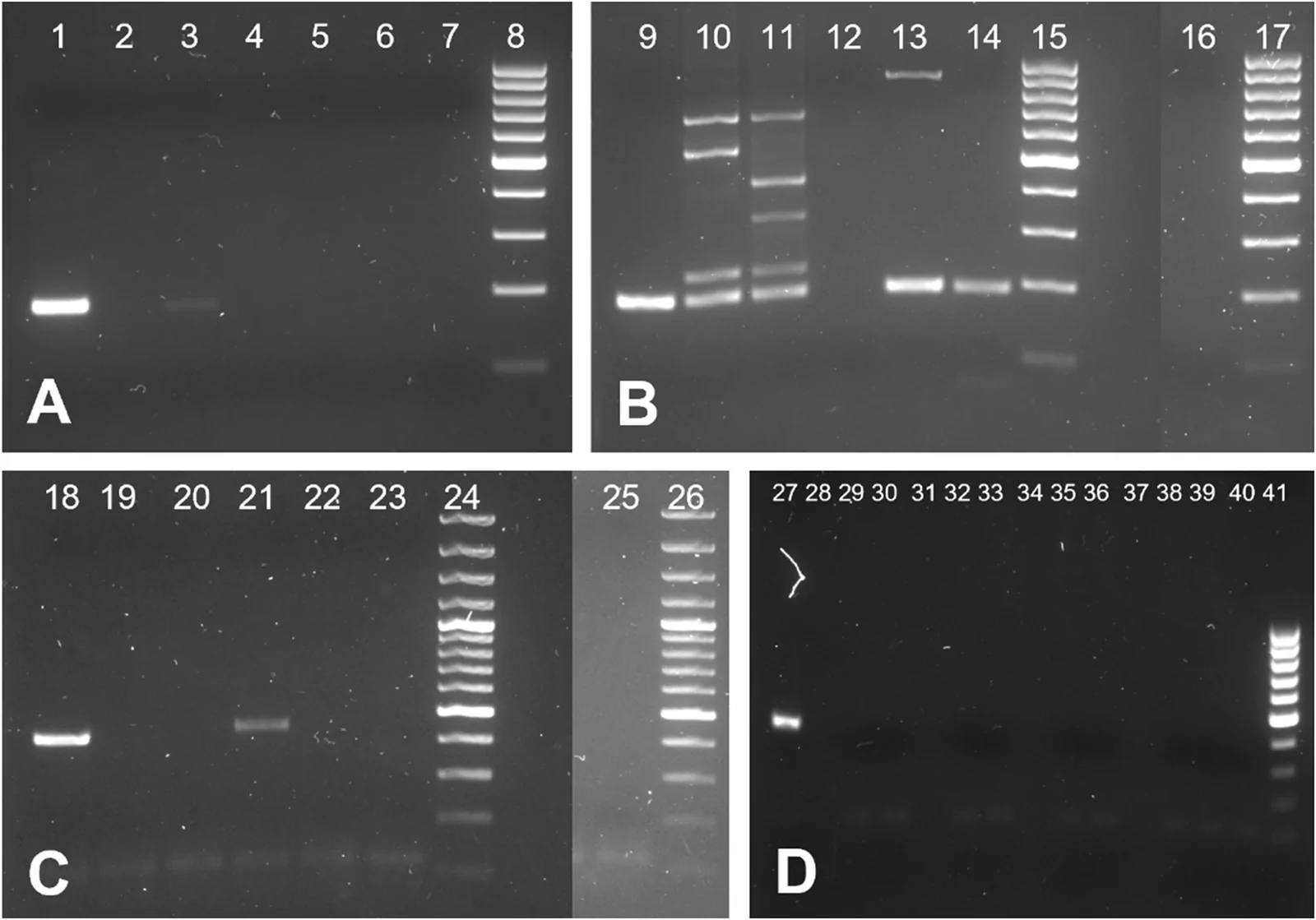
Amplification with *CPN*-16S rRNA primers in Y79 and THP-1 cell gDNA and comparison to *CPN-groL* primers. (A, B) Endpoint PCR with *CPN*-16S rRNA primers. Lane (L)­1: 16S-pUC19 as positive control; L3: Y79 cell gDNA; L4–6: GeneJET Elution Buffer; L7: nontemplate control (NTC); L8, 15, and 17: 100 bp ladder; L9: 16S-pUC19 as positive control; L10–14: THP-1 cell gDNA; L16: NTC. (C) Endpoint PCR with *CPN-groL* primers. L18: gDNA frominfected peripheral blood mononuclear cells as positive control; L19–23 as in L10–14; L24 and 26: 100 bp Plus ladder; L25: NTC. (D) THP-1 cell gDNA quality assessment for *groL*-assays. L27: *groL*-pUC19_3 as positive control; L29–30, 32–33, 35–36, 38–39: THP-1 cell gDNA used for *groL*-assay_3 specifications (technical replicates in neighboring lanes); L40: NTC; L41: 100 bp ladder. All unmentioned lanes are empty.

### *GroL*-Assays

#### Development

For dPCR, a 75–150 bp-amplicon size is recommended because bigger amplicons decrease the mean fluorescence amplitude of positive droplets.^34–36^ In our *C. pneumoniae* primer collection, we found no off-target amplification with the *CPN-groL* primers in four of five THP-1 cell gDNA samples isolated with the GeneJET kit (Figure 5C) and pUC19 DNA (data not shown). Due to their 440 bp-amplicon size, seven primer pairs for the detection of the *C. pneumoniae groL* gene yielding 94–154 bp-amplicons were designed but, despite annealing temperature optimization, found unspecific. Therefore, the impact of the 189- and 440 bp-amplicon sizes on droplet readout was compared in dPCR. For the 16S rRNA amplicon, the mean fluorescence amplitude of positive droplets was 24,858 ± 354 RFU. With 20,603 ± 263 RFU the amplitude decreased by 17.1% for the longer *groL* amplicon (Figure 6A,B). This, however, did not seem to impact the detection of chlamydial DNA (Figure 6C) nor the number of accepted droplets (data not shown): Detected concentrations were 2.5 ± 0.5 and 2.3 ± 0.4 cp/μL, respectively, and the number of accepted droplets was ∼17,000 in both assays.

**Figure 6:**
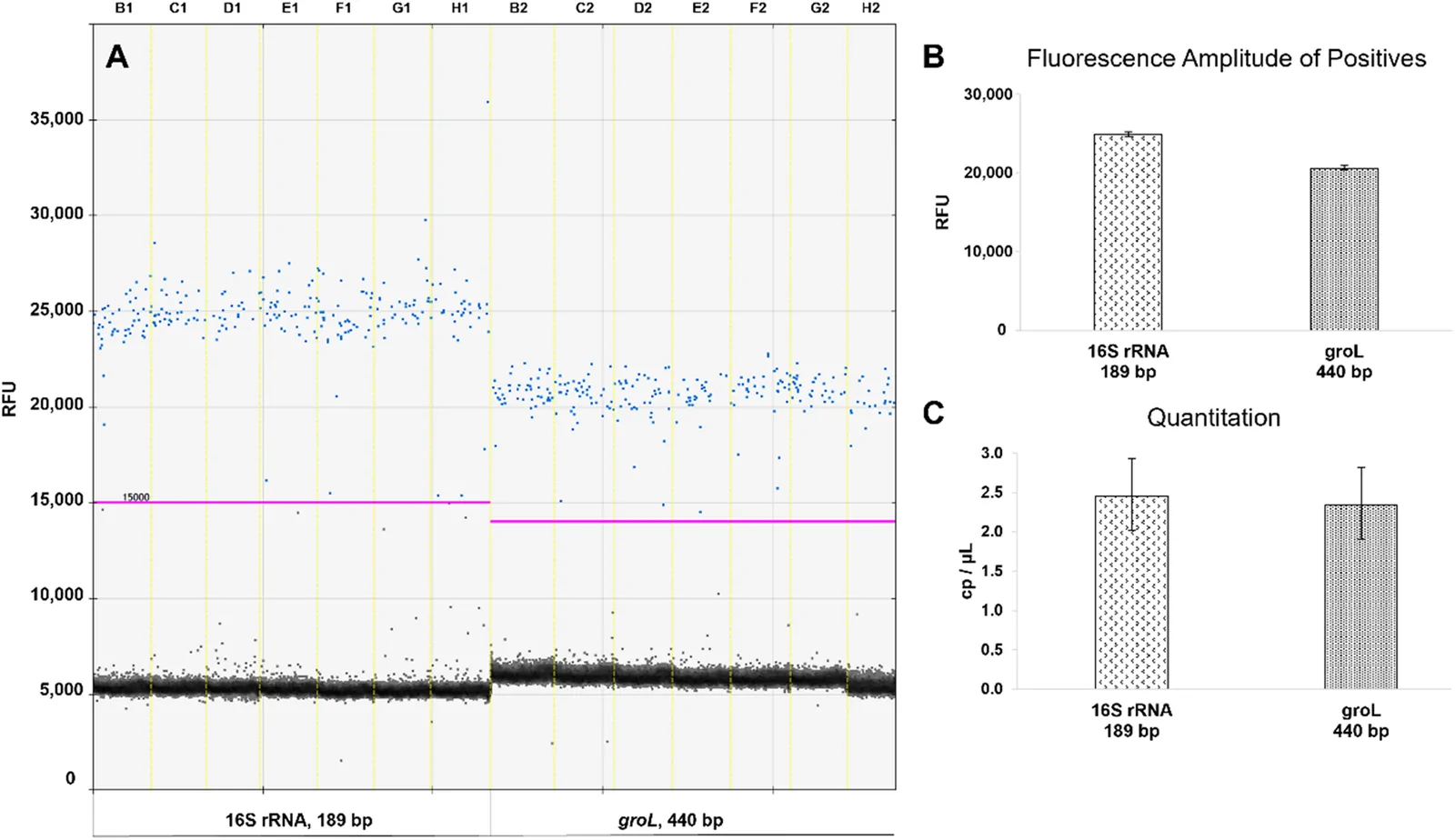
Mean fluorescence amplitude and target quantitation in the 16S- and *groL*-assay_1. (A) Each column in the 1-D amplification plot contains one technical replicate of gDNA extracted from peripheral blood mononuclear cells experimentally infected with *C. pneumoniae* (*n* = 7 technical replicates for each primer). Columns B1–H1: 16S rRNA amplicon, columns B2–H2: *groL* amplicon. Positive droplets show above the indicated threshold, negatives below. Data are presented as mean ± standard deviation for the (B) fluorescence amplitude of positive droplets and (C) target quantitation.

#### Limit of Blank, Limit of Detection, and Limit of Quantification: *groL*-Assay_1

The LOB of the *groL*-assay_1 was with 0.06 cp/μL nearly 5-fold lower than that of the 16S-assay (default thermocycling) and corresponded to 1.20 cp/well (Figure 7A). The LOD was thereafter defined as 0.22 cp/μL (Figure 7B), equaling 4.40 cp/well (Supermix batch 64665535). However, the repeat measurements of LCS were in such a low-concentration range that none resulted in a CV ≤ 25%, which would have been needed to establish the LOQ. Following the EP17-A guideline,^15^ we also decided to include different Supermix lots in our specifications to reflect variability across reagent lots.

**Figure 7:**
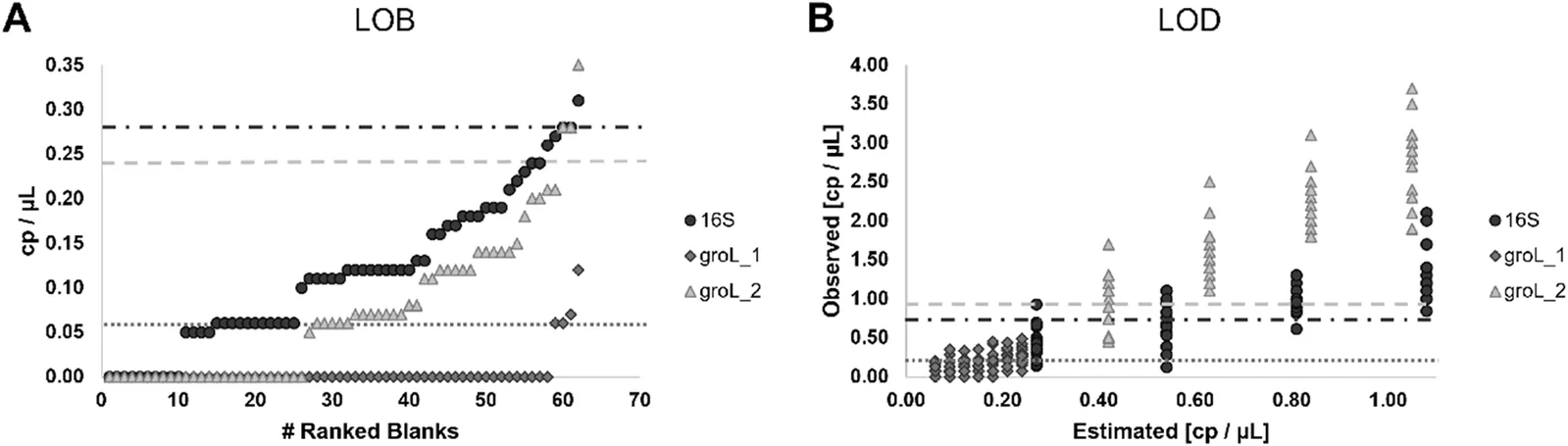
Limit of blank (LOB) and limit of detection (LOD) for the 16S- and the *groL*-assays. The LOBs and LODs are indicated by the dashed lines: dash dot 16S-assay; round dots *groL*-assay_1; dash *groL*-assay_2. Data are presented as single blank measurements or low-concentration samples (LCS) with estimated concentrations. (A) The LOB establishment in the 16S- and the *groL*-assays utilized 63 and 62 blank measurements, respectively. (B) Based on the LOBs, the LODs were established by measuring 15–16 technical replicates per LCS at equidistant concentrations in the range of 0.27–1.08 cp/μL (16S; *n* = 15), 0.06–0.24 cp/μL (*groL*_1; *n* = 16), and 0.42–1.05 cp/μL (*groL*_2; *n* = 15).

#### Limit of Blank, Limit of Detection, and Limit of Quantification: *groL*-Assay_2

To account for interlot differences and to allow complete comparison between d- and nPCR, the *groL*-assay_2 was implemented. It differed regarding the Supermix batch (64679407) and the *groL*-pUC19 plasmid used for specification, as *groL*-pUC19_3 was a slightly extended version of *groL*-pUC19_2, containing annealing sites for the *CPN-groL*_out primers. The LOB for this assay was 0.24 cp/μL (Figure 7A), equaling 4.80 cp/well. The LOD was 0.91 cp/μL (Figure 7B). This translated to 18.2 cp/well. As observed for the 16S-assay (profile “A”) with a factor of 1.9, the lot-to-lot comparison between the *groL*-assays showed an increase of LOB and LOD for the *groL*-assay_2 by a factor of ∼4. This confirmed the assay’s background dependence and consequently the LOD on the Supermix lot. The LOQ was 1.05 cp/μL or 21.0 cp/well.

#### Linearity, Precision, and Trueness: *groL*-Assay_2

In the wells with the highest concentration, all droplets were occupied, rendering concentration estimation based on Poisson distribution impossible. These dilution points were therefore excluded from further analysis and linearity, precision, and trueness calculated for the range 0.1–100,000 cp/well. As recommended,^28^ linearity was assessed using weighted least-squares (WLS) regression only after the quadratic WLS model with HC-3 robust standard errors indicated no deviation from linearity (*p* = 0.115 ns). The WLS regression coefficient *R* ^2^ was 0.996, slope and intercept 1.159 and 0.320, respectively. The precision and trueness estimates are found in Table 4. A relative run-to-run variation lower than the relative repeatability was considered negligible and expressed as 0.0%.^37^ In the range of 100–100,000 cp/well, the assay showed high precision, but with lower concentrations the relative repeatability decreased. The dilution containing 10 cp/well was the closest to the expected value, higher concentrations were overestimated by 13.3–17.5% and lower ones by 37.8–522.2%.

**Table 4:** Precision and Trueness of the *groL*-assay_2^a^

	Measures of Precision			Measures of Trueness	
Dilution	Relative Repeatability [%]	Relative Run-to-Run Variation [%]	Relative Standard Uncertainty [%]	Relative Bias [%]	Recovery [%]
0.1	106.1	61.2	50.0	522.2	622.2
1	128.1	0.0	42.7	37.8	137.8
10	27.7	0.0	9.2	–3.3	96.7
100	7.1	4.3	3.4	17.1	117.1
1000	5.9	0.0	2.0	17.5	117.5
10,000	2.9	1.7	1.4	16.5	116.5
100,000	6.1	0.0	2.0	13.3	113.3

a The terms relative repeatability and intrarun variation essentially describe the same concept and are presented as a measure of method repeatability.

#### Sensitivity Comparison to Quantitative and Nested PCR: *groL*-Assay_1

We subjected technical replicates of the same LCS used for the LOD establishment to nPCR with pUC19 Seq and *CPN-groL*_in as outer and inner primers, respectively. Nested PCR was more sensitive than dPCR. The lowest plasmid concentration reliably detectable was 0.12 cp/μL-PCR reaction, equaling 2.40 cp/reaction (Figure 8B). Through melt-curve comparison in qPCR, it became visible that concentrations as low as 0.06 cp/μL might be detectable, but the detection was unreliable as concentrations of 0.09–0.15 cp/μL remained undetected. The LCS with estimated concentrations of 0.18 and 0.21 cp/μL were reliably detectable (Figure 8E) but 0.24 cp/μL was not, placing the qPCR’s LOD at >0.24 cp/μL.

**Figure 8:**
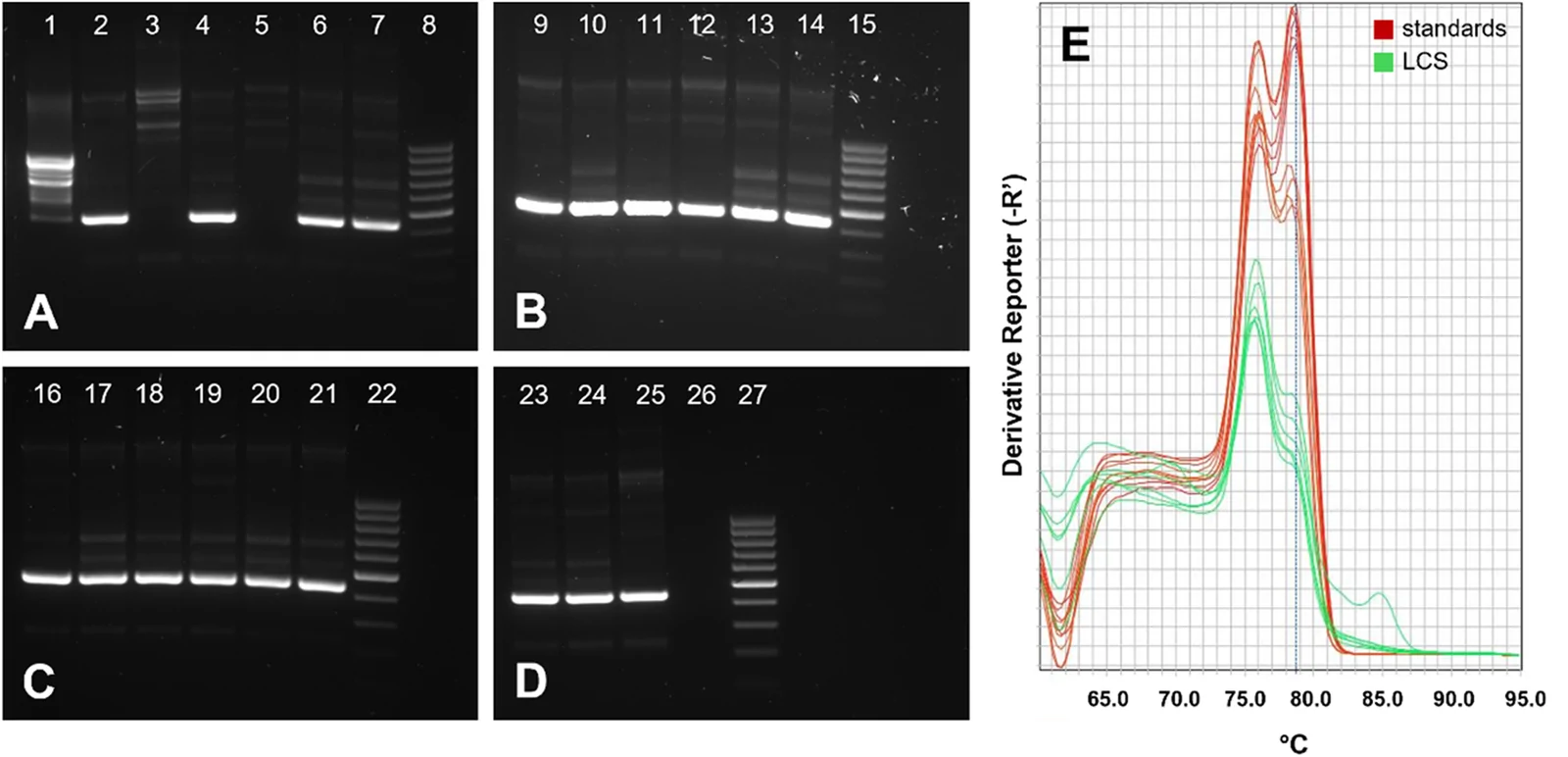
Nested PCR and quantitative (q)­PCR with equidistantly spaced low-concentration samples (LCS) in the range of 0.06–0.24 estimated cp/μL used for limit of detection establishment of the *groL*-assay_1. Each LCS was analyzed in technical triplicate. (A–D) Nested PCR. The outer and inner primers were pUC19 Seq and *CPN-groL*_in, respectively. Lane (L)­1: positive control; L2–4: 0.06 cp/μL; L5–7: 0.09 cp/μL; L9–11: 0.12 cp/μL; L12–14: 0.15 cp/μL; L16–18: 0.18 cp/μL; L19–21: 0.21 cp/μL; L23–25: 0.24 cp/μL; L26: nontemplate control; L8, 15, 22, and 27: 100 bp ladder. (E) Post-qPCR melt-curve analysis of LCS with an estimated concentration of 0.18 and 0.21 cp/μL.

#### Sensitivity Comparison to Quantitative and Nested PCR: *groL*-Assay_2

Nested PCR with *CPN-groL*_out and *CPN-groL*_in as outer and inner primers, respectively, showed reliable amplification across all LCS (Figure 9A,B), undermining the dPCR’s LOD. To determine the lowest concentration detectable in nPCR, a five-point serial dilution of the LCS down to 0.01 cp/μL-PCR reaction in matrix was performed and analyzed in technical triplicates. The lowest reliably detected concentration was 0.05 cp/μL-PCR reaction, equaling 1.00 cp/reaction (Figure 9C,D). In qPCR, concentrations starting from 0.84 cp/μL showed specific melt curves across all technical replicates (Figure 9E). With a dPCR LOD of 0.91 cp/μL-PCR reaction, n- and qPCR were more sensitive, with nPCR in the lead. All assay specifications are summarized in Table 5.

**Table 5:** Digital (d)­PCR Assay Specifications^a^

	16S-Assay	16S-Assay	16S-Assay	*groL*-Assay_1	*groL*-Assay_2
Thermocycling Profile	“A”	“A”	default	default	default
Supermix lot	64639593	64661060	64661060	64665535	64679407
dPCR LOB [cp/μL]/[cp/well]	0.18/3.60	0.35/7.00	0.28/5.60	0.06/1.20	0.24/4.80
dPCR LOD [cp/μL]/[cp/well]	nA	nA	0.74/14.8	0.22/4.40	0.91/18.2
dPCR LOD is at [×LOB]	nA	nA	2.6×	3.7×	3.8×
dPCR LOQ [cp/μL]/[cp/well]	nA	nA	0.81/16.2	>0.24/>4.80	1.05/21.0
lowest LCS detectable in nPCR [cp/μL]/[cp/well]	nA	nA	nA	0.12/2.4	0.05/1.00
lowest LCS detectable in qPCR [cp/μL]/[cp/well]	nA	nA	nA	>0.24/>4.80	0.84/16.8

a **Abbreviations:** LCS low-concentration sample; LOB limit of blank; LOD limit of detection; LOQ limit of quantification; nA not specified; nPCR nested PCR; qPCR quantitative PCR; Supermix QX200 ddPCR EvaGreen Supermix.

**Figure 9:**
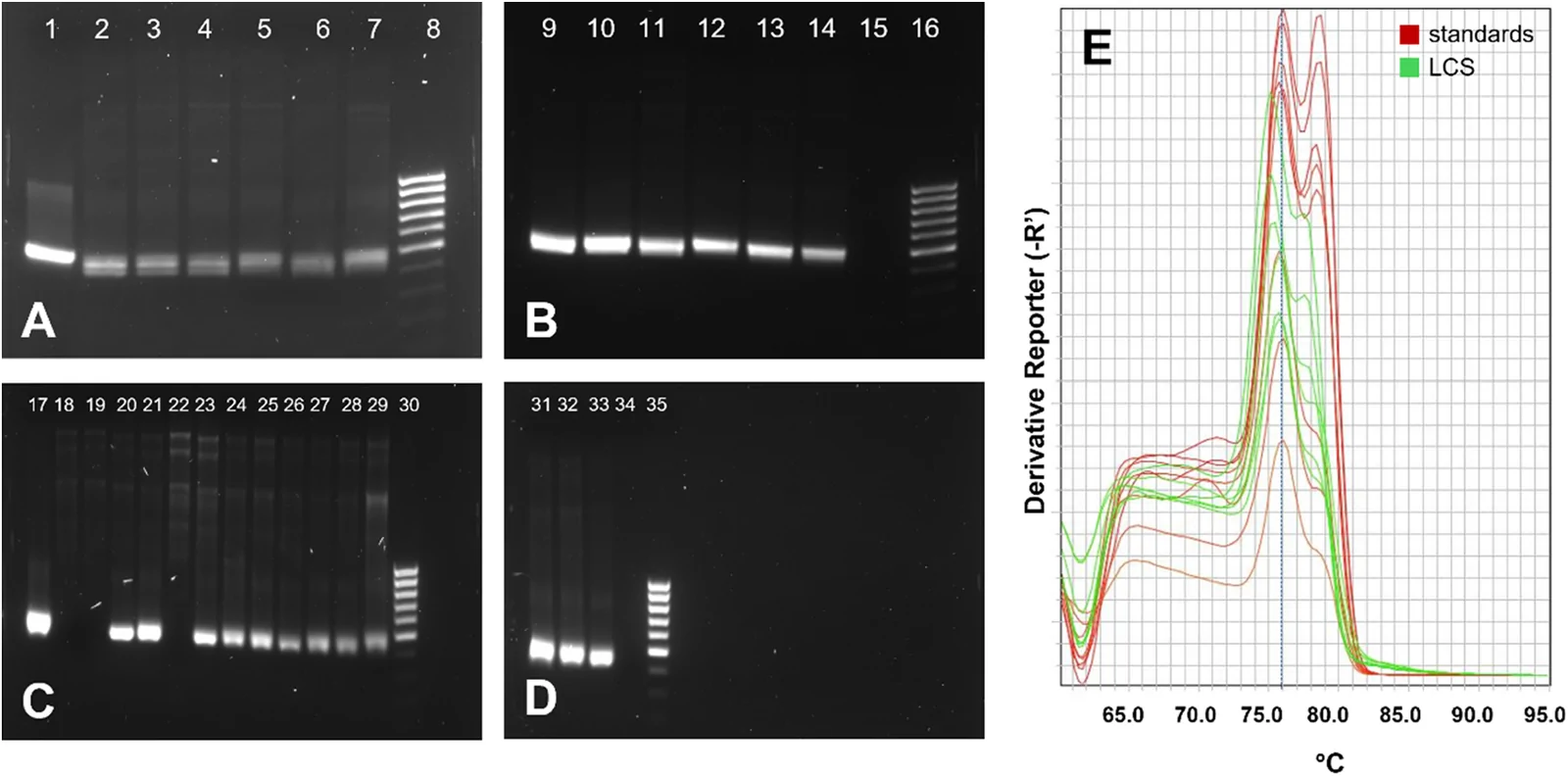
Nested (n)­PCR and quantitative (q)­PCR with equidistantly spaced low-concentration samples (LCS) used for limit of detection (LOD) establishment of the *groL*-assay_2. Each sample was analyzed in technical triplicate, and nPCR was carried out with *CPN-groL*_out and *CPN-groL*_in primers. (A, B) Nested PCR of LCS in the range 0.42–1.05 cp/μL. Lane (L)­1: positive control; L2–4: 0.42 cp/μL; L5–7: 0.63 cp/μL; L8 and 16: 100 bp ladder; L9–11: 0.84 cp/μL; L12–14: 1.05 cp/μL; L15: nontemplate control (NTC). (C, D) Serial dilution of LCS to 0.01–0.21 cp/μL and analysis by nPCR. L17: positive control; L18–20: 0.01 cp/μL; L21–23: 0.03 cp/μL; L24–26: 0.05 cp/μL; L27–29: 0.11 cp/μL; L30 and 35: 100 bp ladder; L31–33: 0.21 cp/μL; L34: NTC. (E) Post-qPCR melt-curve analysis of LCS with an estimated concentration of 0.84 and 1.05 cp/μL.

## Discussion

Highly sensitive PCR tests allow the detection of rare sequences like residuals of pathogen DNA, making them promising candidates as novel diagnostic tools for persistent *C. pneumoniae* infections. Here, we developed and compared two EvaGreen-based dPCR assays for chlamydial detection, and concluded that their LOBs and LODs, as established following the CLSI EP17-A guideline,^15^ were majorly impacted by the Supermix lots. Furthermore, careful evaluation of each assay development step, from primer design to DNA extraction methods and sample storage, proved crucial. Additionally, we present a protocol with example data and calculations for nonparametric LOBs, parametric LODs, LOQs, linearity, precision, and trueness (available as SI).

Digital PCR is praised in many quarters for extraordinary sensitivity, yet most current literature addresses assay development in probe-based applications. DNA-binding dye-based dPCR assays are underrepresented and, to the best of our knowledge, published validation studies of such assays according to the EP17-A guideline^15^ or the ISO 20395:2019 standard^11^ do not exist. DNA-binding dyes offer lower costs, quicker design, and higher flexibility for PCR assays as the same dye can be used with all primers. However, primer specificity is of utmost importance and, although additional probe optimization is not needed, primer optimization can be laborious. High order multiplexing is unfeasible for DNA-binding dye-based analyses unless amplicon lengths or melt curves are considered, which is an important limitation if routine patient examinations would benefit from screening one sample for many possible pathogens simultaneously. In the current application, developing a simple and cost-efficient assay specifically for *C. pneumoniae* detection would not benefit from the multiplexing opportunity. The objective of our study was hence set as providing a thorough validation for a DNA-binding dye-based dPCR method. Many probe-based studies include primer optimization with concentrations ≥ 900 nM.^38–40^ Bio-Rad Laboratories recommend using 100–250 nM primers and ≤ 100 ng DNA input with their EvaGreen-based Supermix. However, the presented application examples do not clearly discern between the chemistries. They rather focus on the outstanding amount of starting material (1 μg/well), enabling detection of a target molecule at an abundance of 0.001%. Together with other probe-based studies,^12,38–40^ this led to the application of the same optimization steps to our DNA-binding dye-based assay. We noticed a significant increase in the negative cluster’s baseline fluorescence with a concomitant higher FPD count when increasing the primer or DNA input. A simple and plausible explanation for this is the ability of the DNA-binding dye to bind to all double-stranded DNA,^41^ no matter if specific amplicon, genome, primer dimers, or heteroduplexes. This elevates the mean fluorescence amplitude of negative droplets and, in extreme cases, results in a high FPD rate or hindrance in partition classification.^42^ So, our DNA-binding dye-based 16S-assay reached its capacity already at <25% of the template input theoretically achievable with probe-based chemistries. This important technical limitation calls for direct comparison of DNA-binding dye-based assays with probe-based assays in future research. Multiple parallel analyses of one sample might mitigate the need for high input DNA per well for a dye-based assay aiming to detect low-abundance targets, but higher costs and small sample quantities might limit practical feasibility of this approach. Our initial strategy to circumvent this bottleneck was to arbitrarily increase target abundance by screening for a sequence repeat in the *C. pneumoniae* gene coding for a DUF1978 domain-containing protein.^43^ However, the repeat length limited primer design with sufficient quality parameters. To the best of our knowledge, the *C. pneumoniae* genome does not contain any multicopy genes; therefore, we concluded to develop an assay detecting the 16S rRNA gene, a frequently used gene for pathogen diagnostics.

When optimizing the 16S-assay, the low recovery was initially attributed to the observed “rain”, which can stem from supercoiled plasmid.^44^ Despite the *Hin*dIII restriction enzyme being explicitly quoted with the Supermix but proving incompatible in our experiments, the enzyme performance might also depend on the supplier or assay itself: Although on a different platform, one earlier study^45^ also reported *Hin*dIII but not *Hae*III or *Mse*I as incapable of digesting 1–2 μg of DNA. We resolved this obstacle by using Supermix-compatible *Sca*I-HF, but the decreasing recovery was not resolved.

DNA concentration measurement methods, subsampling bias, and dead volumes occurring in all steps of the dPCR workflow were addressed as possible contributors to the fluctuating and poor recovery. We first changed the spectrophotometric DNA concentration estimation to fluorometry as the former may overestimate concentrations dependent on the extent of coisolated impurities.^11^ Different DNA isolation kits and sample sources might yield varying degrees of impurities. Thus, it is unsurprising that the factors by which spectrophotometry yields higher values than fluorometry differ between studies and DNA sources. Qubit measurements have been reported to share high concordance with densitometry and qPCR,^46^ but in our study we could not confirm the same for dPCR without important caveats. Indeed, we observed that Qubit quantified significantly less plasmid DNA than NanoDrop, but the dPCR recovery did not improve after adjusting the DNA input accordingly – at least not, when the sample was only measured prestorage. When analyzing the intrarun and -day variability between technical replicates and different samples of sole and spiked-in plasmid, the recovery was stable, indicating a problem different from the instrument or pipetting execution. Given the absence of recovery problems in our previous qPCR studies, we did not use tubes with low-binding properties but stored the DNA in commonly used plastic tubes at −20 °C immediately after isolation. However, apparently large DNA amounts seem to adsorb to the tube walls. Similar observations have been made, where 20–70% plasmid DNA loss occurred within 2–6 weeks of storage at −70 °C even in low-bind tubes,^47^ and 5–95% DNA adsorbed to polypropylene tubes within 3 h.^48^

With sample storage time as the main factor influencing recovery, eventually we found the 16S-assay incompatible with the DNA extraction kit. Nucleic acid contamination in DNA isolation kits is no unknown phenomenon. Among others, the contamination with DNA from *Burkholderia* species,^49^ fungi,^50^ *Legionella*,^50,51^ and *E. coli* (kits used in the Human Genome Project)^52^ has been described. Salter et al.^53^ identified a lot-dependent plethora of bacterial species by sequencing extraction blanks from four kits, among them also unclassified Chlamydiales. Yet possible cross-contamination from our or another laboratory in proximity working with bacteria should neither be neglected even though extensive measures were taken to avoid them. In this regard, the sole amplification of 16S rRNA gene sequences is inconvenient as this gene is highly conserved and amplification might happen in coincidentally present bacteria sharing sufficient homology in the primer binding regions.^54^ Furthermore, an assay based on the amplification of short DNA sequences is more prone to pick up degraded DNA,^55^ as might be derived from contaminated kits. Finally, although NCBI Primer BLAST had indicated specificity for our *CPN*-16S primers, we discovered *in silico* that the same primers also allow the detection of other chlamydial species. Taken together, this led to the assay’s redesign to detect the chlamydial *groL* gene.

In dPCR, short amplicons are preferred^11,12,56^ as longer ones tend to reduce the mean fluorescence amplitude of the positive cluster.^56^ In our experience, however, it was impossible to design specific primers for amplicons ≤ 150 bp, so we settled for a 440 bp-amplicon. Consistent with earlier studies,^34–36,56^ our comparison indicated a reduced mean fluorescence amplitude of the positive cluster, seemingly this phenomenon being independent of the use of probes or DNA-binding dye. Despite this, our assay maintained sensitivity. Aralar et al.^55^ too satisfactorily worked with >600 bp-amplicons when studying post-dPCR melt-curve analyses. To evaluate if the *groL*-assay_1 fulfilled the expectation of dPCR’s outstanding sensitivity, we compared the assay’s performance to q- and nPCR. Quantitative PCR was only marginally and inconsistently more sensitive and melt-curve analysis difficult to interpret for the lowest LCS, but nPCR showed an 1.8-times lower LOD. Since *groL*-pUC19_2 did not contain primer binding sites for the *CPN-groL*_out primers, the first PCR round was conducted with pUC19 Seq primers. Because we could not automatically assume similar efficiency between the pUC19 Seq/*CPN-groL*_in and the *CPN-groL*_out/_in primer combinations but needed to conduct the validation for Supermix lot 64679407 anyway, we decided on *groL*-pUC19 slightly extended with annealing sites for the *CPN-groL*_out primers (*groL*-assay_2). Compared to its equivalent dPCR assay, this nPCR’s LOD was ∼18-times lower. It should be noted that in dPCR the resulting dead volume accumulating from all steps in the dPCR workflow reduces the volume being analyzed, as opposed to 20 μL in nPCR which might contribute to nPCR’s lower LOD. The 2.2-fold difference between both *groL*-assays in nPCR could be due to the different outer primers or to the plasmid extension although no change in secondary structure and only a minimal decrease of –ΔG for the 32 bp-longer plasmid could be observed.

In the existing literature, LOD comparisons between d-, n-, and qPCR are scarce. For *Plasmodium* spp., Mahendran et al.^57^ observed a higher sensitivity for singleplex dPCR than for qPCR. However, duplex qPCR showed higher clinical sensitivity and a better detection of double-infected samples. In their study, nPCR detected more true positive samples based on a different, multicopy, gene than d- or qPCR but lacked in the detection of double-infections. Li et al.^58^ established a dPCR assay for *Bulbus fritillariae cirrhosae* detection with an nPCR-matched LOD of 2.5–5.0 cp/μL. When Voegel et al.^42^ developed their DNA-binding dye-based d- and qPCR assays for soil samples, they found the LODs either to be similar or 10- to 100-fold lower for dPCR. Simplicity and short turnaround times of dPCR together with limited space for samples on a gel in nPCR make nPCR inferior for large-scale studies or routine, high-throughput diagnostics. Susceptibility to carry-over contamination is another important caveat,^24^ and for many applications these limitations might constitute a trade-off between sensitivity and feasibility. Although we found nPCR more sensitive and appreciated the specificity confirmation on the gel, our dPCR assays were still situated in an excellent sensitivity range with LODs of 0.22–0.91 cp/μL or 4.37–18.2 cp/well and the analytical sensitivities well comparable to observations made in probe-based assays: With an LOD of 0.35 cp/μL, a reverse transcription dPCR assay to detect Nipah virus,^59^ showed similar sensitivity as our assays. Seven studies, summarized in a systematic review,^60^ showed LODs between 0.07–34.0 cp/μL for dPCR assays detecting aquaculture pathogens. Another study^28^ compared LODs for bovine papilloma virus detection between different dPCR platforms. The lowest LOD was 6.47 and the highest 45.9 cp/reaction. An alternative approach to LOD estimation in the same study revealed the lowest and highest LOD to be 3.04 and 7.41 cp/reaction.

The LOB, LOD, and LOQ establishment in the presented study was conducted according to the CLSI EP17-A guideline,^15^ a reference especially applicable for clinical chemistry and clinical laboratories. In contrast, the ISO 20395:2019 standard^11^ provides regulations specifically for q- and dPCR to put the more probabilistic nature of PCRs especially at low levels into focus. One important difference between these guidelines lies in the LOD experiments. In the EP17-A approach,^15^ the instrument response for low-level samples with concentrations at the LOB and multiples of it is measured. The LOD is thereafter defined as the lowest concentration reliably distinguishable from the LOB with a certain probability, and its calculation is inherently tied to the LOB. The ISO 20395:2019 standard^11^ describes the LOD establishment explicitly in terms of “probability of detection”. Statistical modeling is based on the detection success among replicate measurements of different concentrations, analyzed in a binary, that is positive–negative, fashion. The lowest concentration in a series of known concentrations with 95% positive replicate measurements for example would correspond to an LOD at 95% confidence. The EP17-A guideline^15^ has been used previously for the validation of probe-based PCR assays.^61–63^ Because this approach incorporates the LOB into the LOD calculation, it provides an appropriate estimate of detection capability especially for assays with nonzero LOBs.^28^ Nevertheless, stochastic effects can influence PCR-based detection especially at minimal target concentrations or LOBs can be extremely low; scenarios for which the approach described in the ISO 20395:2019 standard^11^ will provide a more suitable alternative.

We based other validation parameters, i.e., linearity, precision, and trueness, on the ISO 20395:2019 standard^11^ and obtained an *R* ^2^ higher than the required minimum of >0.99, indicating linearity throughout the measured range of 0.1–100,000 cp/well. Four of seven concentrations yielded smaller relative run-to-run variations than intrarun variations. This might reflect that a major source of variation stems from the assay background rather than differences over time. In contrast to dPCR, Qubit does not have SI-traceability potential, hence the observed bias to overestimate the DNA concentration in all samples but one might be traced back to inaccuracies during initial fluorometric sample measurement or pipetting execution rather than to an inaccurate performance of the dPCR instrument. To circumvent this problem, certified reference material could have been used or the copy number concentration of the initial plasmid stock assessed by dPCR and a trueness/recovery experiment performed based on this value. The latter, however, would have introduced a circular validation problem as measuring dilutions based on the originally dPCR-obtained value would be unable to reveal inaccuracies introduced already during the initial measurement.

We found the LOBs and subsequently the LODs of our *groL*-assay highly dependent on the Supermix lot, which is unproblematic for samples with a high pathogen load as these easily exceed an assay’s LOD. For samples with minute amounts of target, failure to identify an increased background signal while maintaining the previously established LOD will result in false positive calls. In return, keeping an established LOD as cutoff while the background decreases will miss some true positives. Capturing the whole extent of lot-dependent background fluctuation by screening multiple lots and establishing a conservative LOB/LOD avoids constant, laborious, and cost-intensive re-evaluation and troubleshooting, misinterpretation, and repeated analyses, and enables the full potential of low-level detection. In a different approach applicable to small scale studies, Supermix could be obtained in quantities enabling the establishment of one LOB and LOD and conducting a whole study with the same lot number, assuming sufficient shelf life. Alternative approaches like qPCR, whose LOD we found to be almost equal to dPCR, and nPCR, whose LOD surpassed that of dPCR, remain as potentially competitive approaches depending on the study framework.

## Conclusions

This study shows that in the QX200 Droplet Digital PCR system the assay’s LOB and LOD are dependent on the Supermix lot. This has implications for large scale studies and routine high-throughput diagnostics, because it hampers the detection of minute target amounts and calls either for a very conservative LOD or its re-establishment upon lot change. To avoid low recovery rates, optimization of sample storage procedures by e.g., using carrier DNA or freshly isolated sample or the constant fluorometric reassessment of DNA concentrations may be required.

## Supplementary Material







## Notes

SI_dMIQE Checklist. Detailed description of experimental parameters for dPCR (XLSX)
SI_LCS Calculations. An example calculation for a set of low-concentration samples in the *groL*-assay_1 (PDF)
SI_SI Tables 1–6. Raw data and calculations for limit of blank, limit of detection, limit of quantification, linearity, precision, and trueness, as well as all primer sequences (XLSX)

## References

[1] Saiki R. K., Scharf S., Faloona F., Mullis K. B., Horn G. T., Erlich H. A., Arnheim N. (1985). Enzymatic Amplification of Beta-Globin
Genomic Sequences and Restriction Site Analysis for Diagnosis of Sickle
Cell Anemia. Science.

[2] Mullis, K. B. ; Faloona, F. A. Specific Synthesis of DNA in Vitro via a Polymerase-Catalyzed Chain Reaction. In Methods in Enzymology 1987; Vol. 155, pp 335–350. 10.1016/0076-6879(87)55023-6 3431465

[3] Saiki R. K., Gelfand D. H., Stoffel S., Scharf S. J., Higuchi R., Horn G. T., Mullis K. B., Erlich H. A. (1988). Primer-Directed
Enzymatic Amplification of DNA with a Thermostable DNA Polymerase. Science.

[4] Luthra, R. ; Singh, R. R. ; Patel, K. P. Preface. In Clinical Applications of PCR, 3rd ed.; Luthra, R. ; Singh, R. R. ; Patel, K. P. , Eds.; Methods in Molecular Biology 1392; Humana Press: New York, N.Y., 2016; p V.

[5] Ruano G., Kidd K. K., Stephens J. C. (1990). Haplotype
of multiple polymorphisms
resolved by enzymatic amplification of single DNA molecules. Proc. Natl. Acad. Sci. U.S.A..

[6] Vogelstein B., Kinzler K. W. (1999). Digital PCR. Proc. Natl. Acad.
Sci. U.S.A..

[7] Heid C. A., Stevens J., Livak K. J., Williams P. M. (1996). Real Time Quantitative
PCR. Genome Res..

[8] Ruijter J. M., van den Hoff M. J. B. (2025). Analysis
of qPCR Data: From PCR Efficiency to Absolute
Target Quantity. Int. J. Mol. Sci..

[9] Svec D., Tichopad A., Novosadova V., Pfaffl M. W., Kubista M. (2015). How good is
a PCR efficiency estimate: Recommendations for precise and robust
qPCR efficiency assessments. Biomol. Detect.
Quantif..

[10] Reischl U. (2006). Melting of
the Ribosomal RNA Gene Reveals Bacterial Species Identity: A Step
toward a New Rapid Test in Clinical Microbiology. Clin. Chem..

[11] International Organization for Standardization ISO 20395:2019 Biotechnology - Requirements for Evaluating the Performance of Quantification Methods for Nucleic Acid Target Sequences - QPCR and DPCR. Geneva, Switzerland, 2019.

[12] Witte A. K., Mester P., Fister S., Witte M., Schoder D., Rossmanith P. (2016). A Systematic Investigation of Parameters
Influencing
Droplet Rain in the Listeria Monocytogenes prfA Assay - Reduction
of Ambiguous Results in DdPCR. PLoS One.

[13] Majumdar N., Banerjee S., Pallas M., Wessel T., Hegerich P. (2017). Poisson Plus
Quantification for Digital PCR Systems. Sci.
Rep..

[14] Huggett J. F., Cowen S., Foy C. A. (2015). Considerations
for Digital PCR as
an Accurate Molecular Diagnostic Tool. Clin.
Chem..

[15] The National Committee for Clinical Laboratory Standards . EP17-A – Protocols for Determination of Limits of Detection and Limits of Quantitation; Approved Guideline; Wayne: P.A., 2004; Vol. 24, p 34.

[16] Dehghani M., Norouzi H., Dehghan S., Spruill M. L., Goodarzi M., Lee K. H., Martin H. L. (2026). Comparative
diagnostic evaluation
of real-time PCR and culture for detecting pathogens in podiatric
wound infections. Microbiol. Spectr..

[17] Kömeç S., Durmuş M. A., Ceylan A. N., Korkusuz R. (2025). A Novel PCR Panel for
Bacterial Detection in Lower Respiratory Tract Infections: A Comparative
Study with Culture Results. Pathogens.

[18] Sun J., Zhao S., Wei C., Liu J., Chen J., Xing L., Yan H., Zhang Y., Bai R., Zhang Z. (2022). A diagnostic test of real-time PCR detection in the
diagnosis of
clinical bloodstream infection. Ann. Palliat.
Med..

[19] Tagini F., Puolakkainen M., Greub G. (2025). From coughs to complications: The
story of Chlamydia pneumoniae. J. Med. Microbiol..

[20] U.S. Centers for Disease Control and Prevention . Clinical Features of Chlamydia pneumoniae Infection. https://www.cdc.gov/cpneumoniae/hcp/clinical-signs/ (accessed 2026–05–19).

[21] Centre for Biosecurity of the Public Health Agency of Canada . Chlamydophila pneumoniae: Infectious substance pathogen safety data sheet. https://www.canada.ca/en/public-health/services/laboratory-biosafety-biosecurity/pathogen-safety-data-sheets-risk-assessment/chlamydophila-pneumoniae.html (accessed 2026–05–19).

[22] Porritt R. A., Crother T. R. (2016). Chlamydia pneumoniae Infection and Inflammatory Diseases. For. Immunopathol. Dis. Therap..

[23] U.S. Centers for Disease Control and Prevention . Laboratory Testing for Chlamydia pneumoniae. https://www.cdc.gov/cpneumoniae/php/laboratories/index.html(accessed 2026–06–10).

[24] Dowell S. F., Peeling R. W., Boman J., Carlone G. M., Fields B. S., Guarner J., Hammerschlag M. R., Jackson L. A., Kuo C.-C., Maass M., Messmer T. O., Talkington D. F., Tondella M. L., Zaki S. R. (2001). Standardizing Chlamydia pneumoniae
Assays: Recommendations from the Centers for Disease Control and Prevention
(USA) and the Laboratory Centre for Disease Control (Canada). Clin. Infect. Dis..

[25] Maass M., Bartels C., Engel P. M., Sievers H.-H. (1998). Endovascular Presence
of Viable Chlamydia pneumoniae Is a Common Phenomenon in Coronary
Artery Disease. J. Am. Coll. Cardiol..

[26] Kortesoja M., Trofin R. E., Hanski L. (2020). A platform for studying the transfer
of Chlamydia pneumoniae infection between respiratory epithelium and
phagocytes. J. Microbiol. Methods.

[27] Kralik P., Ricchi M. (2017). A Basic Guide to Real
Time PCR in Microbial Diagnostics:
Definitions, Parameters, and Everything. Front.
Microbiol..

[28] Gleerup D., Vynck M., Gysens L., De Baere C., Trypsteen W., Vandesompele J., Thas O., Martens A., Haspeslagh M., De Spiegelaere W. (2026). Standardized digital PCR assay validation
using PCR-ValiPal,
demonstrated in cross-platform quantification of bovine papilloma
virus. Anal. Chim. Acta.

[29] Reigada I., Kapp K., Kaudela T., García Soria M., Oksanen T., Hanski L. (2024). Tracking Chlamydia
– Host
interactions and antichlamydial activity in Caenorhabditis elegans. Biomed. Pharmacother..

[30] Berger M., Schröder B., Daeschlein G., Schneider W., Busjahn A., Buchwalow I., Luft F. C., Haller H. (2000). Chlamydia
pneumoniae DNA in non-coronary atherosclerotic plaques and circulating
leukocytes. J. Lab. Clin. Med..

[31] Ikejima H., Friedman H., Leparc G. F., Yamamoto Y. (2005). Depletion of Resident
Chlamydia pneumoniae through Leukoreduction by Filtration of Blood
for Transfusion. J. Clin. Microbiol..

[32] Haranaga S., Yamaguchi H., Leparc G. F., Friedman H., Yamamoto Y. (2001). Detection
of Chlamydia pneumoniae antigen in PBMNCs of healthy blood donors. Transfusion.

[33] Reinhold P., Sachse K., Kaltenboeck B. (2011). Chlamydiaceae
in cattle: Commensals,
trigger organisms, or pathogens?. Vet. J..

[34] Laurie M. T., Bertout J. A., Taylor S. D., Burton J. N., Shendure J. A., Bielas J. H. (2013). Simultaneous Digital Quantification and Fluorescence-Based
Size Characterization of Massively Parallel Sequencing Libraries. BioTechniques.

[35] Miotke L., Lau B. T., Rumma R. T., Ji H. P. (2014). High Sensitivity
Detection and Quantitation of DNA Copy Number and Single Nucleotide
Variants with Single Color Droplet Digital PCR. Anal. Chem..

[36] McDermott G. P., Do D., Litterst C. M., Maar D., Hindson C. M., Steenblock E. R., Legler T. C., Jouvenot Y., Marrs S. H., Bemis A., Shah P., Wong J., Wang S., Sally D., Javier L., Dinio T., Han C., Brackbill T. P., Hodges S. P., Ling Y., Klitgord N., Carman G. J., Berman J. R., Koehler R. T., Hiddessen A. L., Walse P., Bousse L., Tzonev S., Hefner E., Hindson B. J., Cauly T. H., Hamby K., Patel V. P., Regan J. F., Wyatt P. W., Karlin-Neumann G. A., Stumbo D. P., Lowe A. J. (2013). Multiplexed target detection using
DNA-binding dye chemistry in droplet digital PCR. Anal. Chem..

[37] Deprez L., Corbisier P., Kortekaas A. M., Mazoua S., Beaz Hidalgo R., Trapmann S., Emons H. (2016). Validation of a digital PCR method
for quantification of DNA copy number concentrations by using a certified
reference material. Biomol. Detect. Quantif..

[38] Gerdes L., Iwobi A., Busch U., Pecoraro S. (2016). Optimization of digital
droplet polymerase chain reaction for quantification of genetically
modified organisms. Biomol. Detect. Quantif..

[39] Long S., Berkemeier B. (2020). Development
and optimization of a simian immunodeficiency
virus (SIV) droplet digital PCR (ddPCR) assay. PLoS One.

[40] Maier J., Lange T., Cross M., Wildenberger K., Niederwieser D., Franke G. N. (2019). Optimized Digital Droplet PCR for
BCR-ABL. J. of Mol. Diagn..

[41] Mao F., Leung W. Y., Xin X. (2007). Characterization
of EvaGreen and
the implication of its physicochemical properties for qPCR applications. BMC Biotechnol..

[42] Voegel T. M., Larrabee M. M., Nelson L. M. (2021). Development
of droplet digital PCR
assays to quantify genes involved in nitrification and denitrification,
comparison with quantitative real-time PCR and validation of assays
in vineyard soil. Can. J. Microbiol..

[43] Rocha E. P. C., Pradillon O., Bui H., Sayada C., Denamur E. (2002). A new family
of highly variable proteins in the Chlamydophila pneumoniae genome. Nucleic Acids Res..

[44] Beinhauerova M., Babak V., Bertasi B., Boniotti M. B., Kralik P. (2020). Utilization
of Digital PCR in Quantity Verification of Plasmid Standards Used
in Quantitative PCR. Front. Mol. Biosci..

[45] Kang Q., Parkin B., Giraldez M. D., Tewari M. (2016). Mutant DNA
Quantification
by Digital PCR Can Be Confounded by Heating During DNA Fragmentation. BioTechniques.

[46] Simbolo M., Gottardi M., Corbo V., Fassan M., Mafficini A., Malpeli G., Lawlor R. T., Scarpa A. (2013). DNA Qualification Workflow
for Next Generation Sequencing of Histopathological Samples. PLoS One.

[47] Wang Y., Keith M., Leyme A., Bergelson S., Feschenko M. (2019). Monitoring long-term DNA storage via absolute copy
number quantification by ddPCR. Anal. Biochem..

[48] Gaillard C., Strauss F. (1998). Avoiding adsorption
of DNA to polypropylene tubes and
denaturation of short DNA fragments. Technol.
Tips Online.

[49] Peters R. P. H., Mohammadi T., Vandenbroucke-Grauls C. M. J. E., Danner S. A., Van Agtmael M. A., Savelkoul P. H. M. (2004). Detection
of bacterial DNA in blood samples from febrile patients: underestimated
infection or emerging contamination?. FEMS Immunol.
Med. Microbiol..

[50] Evans G. E., Murdoch D. R., Anderson T. P., Potter H. C., George P. M., Chambers S. T. (2003). Contamination of Qiagen DNA Extraction
Kits with Legionella
DNA. J. Clin. Microbiol..

[51] van
der Zee A., Peeters M., de Jong C., Verbakel H., Crielaard J. W., Claas E. C. J., Templeton K. E. (2002). Qiagen
DNA extraction kits for sample preparation for Legionella PCR are
not suitable for diagnostic purposes. J. Clin.
Microbiol..

[52] Glassing A., Dowd S. E., Galandiuk S., Davis B., Chiodini R. J. (2016). Inherent
bacterial DNA contamination of extraction and sequencing reagents
may affect interpretation of microbiota in low bacterial biomass samples. Gut Pathog..

[53] Salter S. J., Cox M. J., Turek E. M., Calus S. T., Cookson W. O., Moffatt M. F., Turner P., Parkhill J., Loman N. J., Walker A. W. (2014). Reagent and laboratory
contamination can critically
impact sequence-based microbiome analyses. BMC
Biol..

[54] Clarridge J. E., 3rd (2004). Impact of 16S rRNA
gene sequence analysis for identification of bacteria on clinical
microbiology and infectious diseases. Clin.
Microbiol. Rev..

[55] Aralar A., Goshia T., Ramchandar N., Lawrence S. M., Karmakar A., Sharma A., Sinha M., Pride D. T., Kuo P., Lecrone K., Chiu M., Mestan K. K., Sajti E., Vanderpool M., Lazar S., Crabtree M., Tesfai Y., Frayley S. I. (2024). Universal
digital high-resolution melt analysis for
the diagnosis of bacteremia. J. Mol. Diagn..

[56] Krumbholz M., Goerlitz K., Albert C., Lawlor J., Suttorp M., Metzler M. (2019). Large amplicon droplet
digital PCR for DNA-based monitoring
of pediatric chronic myeloid leukaemia. J. Cell.
Mol. Med..

[57] Mahendran P., Liew J. W. K., Amir A., Ching X. T., Lau Y. L. (2020). Droplet
digital polymerase chain reaction (ddPCR) for the detection of Plasmodium
knowlesi and Plasmodium vivax. Malar. J..

[58] Li Y., Hu J., Chen H., Zhao T., Niu S., Liu S., Bai J., Chen R., Guo J. (2026). Comparison of nested PCR coupled
DNA barcoding and an all-in-one dd PCR in detection of highly degraded
DNA in Fritillariae Cirrhosae Bulbus. Microchem.
J..

[59] Shuai J., Chen K., Han X., Zeng R., Song H., Fu L., Zhang X. (2024). Development
and validation of a droplet digital PCR
assay for Nipah virus quantitation. BMC Vet.
Res..

[60] Sumon M. A. A., Meregildo-Rodriguez E. D., Lee P. T., Dinh-Hung N., Larson E. T., Permpoonpattana P., Van Doan H., Jung W. K., Linh N. V. (2024). Droplet digital
PCR for fish pathogen detection and
quantification: A systematic review and meta-analysis. J. Fish Dis..

[61] Milbury C. A., Zhong Q., Lin J., Williams M., Olson J., Link D. R., Hutchison B. (2014). Determining Lower Limits of Detection
of Digital PCR Assays for Cancer-Related Gene Mutations. Biomol. Detect. Quantif..

[62] Liu Y., Han C., Li J., Xu S., Xiao Z., Guo Z., Rao S., Yao Y. (2024). Laboratory-developed
Droplet Digital PCR Assay for
Quantification of the JAK^2V617F^ Mutation. Glob. Med. Genet..

[63] Zhao Y., Yan Z., Song K., Li Y., Shen L., Cui Y., Du Z., Yang R., Song Y., Jing L., Zhao Y. (2024). Development
and evaluation of a multi-target droplet digital PCR assay for highly
sensitive and specific detection of Yersinia pestis. PLoS Negl. Trop. Dis..

