# Supplementary material for: Limit of Blank Variability as a Determinant of Analytical Sensitivity in a DNA-Binding Dye-Based Digital Polymerase Chain Reaction for the Detection of Chlamydia pneumoniae
Source: Anal Chem. 2026 Sep 4;98(36):27053–67. doi: 10.1021/acs.analchem.6c04206 (PMC13584697; doi:10.1021/acs.analchem.6c04206)
Supplement: Supplementary file 1 [file ac6c04206_si_002.pdf]

# Limit of Blank Variability as a Determinant of Analytical Sensitivity in a DNA-Binding Dye-Based Digital Polymerase Chain Reaction for the Detection of *Chlamydia pneumoniae*

*Theresa Kaudela, Eveliina Taavitsainen-Wahlroos, and Leena Hanski**

Division of Pharmaceutical Biosciences, Faculty of Pharmacy, University of Helsinki, 00014 Helsinki, Finland

**Table of Contents**

**SI_dMIQE Checklist.** (available in the Excel file "SI_dMIQE Checklist")
**SI Table 1.** Limit of blank (LOB) estimation for all assays. (available in the Excel file "SI Tables 1–6")
**Example calculations for low-concentration samples (LCS) for the *groL*-assay_1**
**SI Table 2.** Limit of detection (LOD) and limit of quantification (LOQ) estimation for the 16S-assay. (available in the Excel file "SI Tables 1–6")
**SI Table 3.** Limit of detection (LOD) and limit of quantification (LOQ) estimation for the *groL*-assay_1. (available in the Excel file "SI Tables 1–6")
**SI Table 4.** Limit of detection (LOD) and limit of quantification (LOQ) estimation for the *groL*-assay_2. (available in the Excel file "SI Tables 1–6")
**SI Table 5.** Linearity, precision, and accuracy estimation for the *groL*-assay_2. (available in the Excel file "SI Tables 1–6")
**SI Table 6.** Primer sequences. (available in the Excel file "SI Tables 1–6")

**Example calculations for low-concentration samples (LCS) for the *groL*-assay_1**

***groL*-assay_1 <u>preliminary</u> limit of blank (LOB):** 0.027 cp/µL or 0.540 copies per 20 µL-well (**note:** in the manuscript, the LCS are presented as multiples of 0.03 cp/µL, resulting in a range of 0.06–0.24 cp/µL in the final dPCR reaction)

**LCS concentrations:** 0.06–0.24 cp/µL PCR reaction, frozen as single-use aliquots

***groL*-pUC19_2 DNA:** 4.2 ng/µL

***groL*-pUC19_2 size:** 3,314 bp
**molar mass per base pair:** 650 (g/mol)/bp (default)

$$groL\_pUC19\_2\ [ng/mol] = (3\ 314\ bp \times 650\ (g/mol)/bp) \times 10^9$$

$$groL\_pUC19\_2 = 2.15 \times 10^{15}\ ng/mol$$

If 1 bp equals 650 g/mol, 3 314 bp equal $2.15 \times 10^6$ g/mol or $2.15 \times 10^{15}$ ng/mol.

$$2.15 \times 10^{15}\ ng \ldots 1\ mol$$
$$4.2\ ng \ldots\ 1.95 \times 10^{-15} mol$$

$$grol\_pUC19\_2\ stock\ [mol/\mu L] = \frac{4.2\ ng/\mu L}{2.15 \times 10^{15}\ ng/mol}$$

$$grol\_pUC19\_2\ stock = 1.95 \times 10^{-15}\ mol/\mu L$$

If $2.15 \times 10^{15}$ ng equal 1 mol, a plasmid stock concentration of 4.2 ng/µL equals $1.95 \times 10^{-15}$ mol/µL.

**Avogadro's number:** $6.022 \times 10^{23}$ cp/mol

$$groL\_pUC19\_2\ stock\ [cp/\mu L] = 1.95 \times 10^{-15}\ mol/\mu L \times 6.022 \times 10^{23} cp$$

$$groL\_pUC19\_2\ stock = 1.17 \times 10^9\ cp/\mu L$$

If 1 mol equals $6.022 \times 10^{23}$ cp, $1.95 \times 10^{-15}$ mol/µL equal $1.17 \times 10^9$ cp/µL. Concretely, the number is 1 174 151 618 cp/µL.

To create a working stock concentration, *groL*-pUC19_2 was diluted 1:1 000 and this dilution 1:1 000 too, resulting in 1 174 cp/µL. This 1 174 cp/µL-dilution was diluted to 3 cp/µL in 500 µL.

$$V_1 of\ groL\_pUC19\_2 = \frac{3\ cp/\mu L \times 500\ \mu L}{1\ 174\ cp/\mu L}$$

$$V_1 = 1.28\ \mu L\ groLpUC19\_2 + 498.72\ \mu L\ NFW$$

**One 20 µL-well / 22 µL-well contains:**
125 / 137.5 ng THP-1 cell gDNA as matrix
*groL*-pUC19_2 in varying concentrations (1.08–4.32 cp per 20 µL-well or 1.19–4.75 cp per 22 µL-well)

**THP-1 cell gDNA:** 52.6 ng/µL

**To create LCS matrix for one 22 µL-well:**

$$\mu L\ THP1\ cell\ gDNA = \frac{137.5\ ng}{52.6\ ng/\mu L}$$

**example for LCS #1 (for one 22 µL-well):**

$$\mu L\ grol\_pUC19\_2 = \frac{1.19\ cp}{3\ cp/\mu L}$$

| **LCS<br>[cp per 20 µL-well]<br>[cp per 22 µL-well]** | **#1<br>1.08<br>1.19** | **#2<br>1.62<br>1.78** | **#3<br>2.16<br>2.38** | **#4<br>2.70<br>2.97** | **#5<br>3.24<br>3.56** | **#6<br>3.78<br>4.16** | **#7<br>4.32<br>4.75** |
|---|---|---|---|---|---|---|---|
| **THP-1 cell DNA [µL]** | 2.61 | 2.61 | 2.61 | 2.61 | 2.61 | 2.61 | 2.61 |
| ***groL*-pUC19_2 [µL]** | 0.40 | 0.59 | 0.79 | 0.99 | 1.19 | 1.39 | 1.58 |

To create a single-use aliquot for one dPCR run á 4 wells per LCS (including 1 well surplus for pipetting loss) the depicted amount would be needed:

| | **THP-1 cell gDNA (52.6 cp/µL)** | ***groL*-pUC19_2 (3 cp/µL)** |
|---|---|---|
| **#1** | 13.07 | 1.98 |
| **#2** | 13.07 | 2.97 |
| **#3** | 13.07 | 3.96 |
| **#4** | 13.07 | 4.95 |
| **#5** | 13.07 | 5.94 |
| **#6** | 13.07 | 6.93 |
| **#7** | 13.07 | 7.92 |

To ensure that all four dPCR runs needed for the specification of the limit of detection (LOD) have reduced variability stemming from pipetting, a stock for eight runs was created and aliquoted into single-use aliquots:

| | **THP-1 cell gDNA (52.6 cp/µL)** | ***groL*-pUC19_2 (3 cp/µL)** | **aliquot á [µL]** | **added per 22 µL-well [µL]** |
|---|---|---|---|---|
| **#1** | 104.6 | 15.84 | 15.05 | 3.01 |
| **#2** | 104.6 | 23.73 | 16.04 | 3.21 |
| **#3** | 104.6 | 31.68 | 17.03 | 3.41 |
| **#4** | 104.6 | 39.60 | 18.02 | 3.60 |
| **#5** | 104.6 | 47.52 | 19.01 | 3.80 |
| **#6** | 104.6 | 55.44 | 20.00 | 4.00 |
| **#7** | 104.6 | 63.36 | 20.99 | 4.20 |
